# Population Genomic Analysis of 962 Whole Genome Sequences of Humans Reveals Natural Selection in Non-Coding Regions

**DOI:** 10.1371/journal.pone.0121644

**Published:** 2015-03-25

**Authors:** Fuli Yu, Jian Lu, Xiaoming Liu, Elodie Gazave, Diana Chang, Srilakshmi Raj, Haley Hunter-Zinck, Ran Blekhman, Leonardo Arbiza, Cris Van Hout, Alanna Morrison, Andrew D. Johnson, Joshua Bis, L. Adrienne Cupples, Bruce M. Psaty, Donna Muzny, Jin Yu, Richard A. Gibbs, Alon Keinan, Andrew G. Clark, Eric Boerwinkle

**Affiliations:** 1 Human Genome Sequencing Center, Molecular and Human Genetics Department, Baylor College of Medicine, Houston, Texas, United States of America; 2 Department of Molecular Biology and Genetics, Cornell University, Ithaca, New York, New York, United States of America; 3 Human Genetic Center, University of Texas Health Science Center, Houston, Texas, United States of America; 4 National Heart, Lung and Blood Institute (NHLBI) Framingham Heart Study, Framingham, Massachusetts, United States of America; 5 Cardiovascular Health Research Unit, Departments of Medicine, Epidemiology, and Health Services, University of Washington, Seattle, Washington, United States of America; 6 Institute of Neurology, Tianjin Medical University General Hospital, Tianjin, China; 7 College of Life Sciences, State Key Laboratory of Protein and Plant Gene Research, Center for Bioinformatics, Peking-Tsinghua Center for Life Sciences, Peking University, Beijing, China; National Institutes of Health, UNITED STATES

## Abstract

Whole genome analysis in large samples from a single population is needed to provide adequate power to assess relative strengths of natural selection across different functional components of the genome. In this study, we analyzed next-generation sequencing data from 962 European Americans, and found that as expected approximately 60% of the top 1% of positive selection signals lie in intergenic regions, 33% in intronic regions, and slightly over 1% in coding regions. Several detailed functional annotation categories in intergenic regions showed statistically significant enrichment in positively selected loci when compared to the null distribution of the genomic span of ENCODE categories. There was a significant enrichment of purifying selection signals detected in enhancers, transcription factor binding sites, microRNAs and target sites, but not on lincRNA or piRNAs, suggesting different evolutionary constraints for these domains. Loci in “repressed or low activity regions” and loci near or overlapping the transcription start site were the most significantly over-represented annotations among the top 1% of signals for positive selection.

## Introduction

Identifying genomic regions whose patterns of polymorphism are consistent with the past action of natural selection has been very fruitful in recent years[1–13]. Despite the success, three primary caveats continue to bear on these studies: (1) Ascertainment of variants using high-throughput genotyping assays (such as the International HapMap Project[11]) were predominantly biased to the common portion of the site frequency spectrum (SFS), which probably did not significantly affect the positive selection signals using haplotype-based approaches (such as EHH[14] and iHS[15]) as much as it would reduce the power for SFS tests especially when screening for negatively selected loci; (2) Sample size from any one ethnic group were low (low hundreds), further reducing the power for fitting models of demography and selection; and (3) The annotations in the noncoding regions have been too sparse to adequately motivate empirical tests to corroborate the detected signals or perform further follow-up studies.

A number of exciting developments in the past 2–3 years provide new opportunities to address the caveats listed above. The application of NGS to interrogate a large number of samples in cohort studies has become increasingly feasible[1,3,5], and ENCODE studies[16–18] and informatics tools[19] have provided functional implications in canonically intergenic sequences. Further application of population genetic principles and statistical tests to these large-scale sequencing datasets with their improved functional annotations holds promise to reveal the evolutionary and biological processes that have shaped patterns of variation in the human genome.

In this study, we report our analysis of 962 whole genome sequences of European Americans, who were primarily enrolled for prospective epidemiology studies[20]. The genotype-phenotype association studies have been published previously[21]. This dataset of large sample from one ethnic group serves as a unique resource for understanding the metrics on rare variants and their implications for both population and human genetics studies. In our study, we identified a large number of loci under both positive and negative selection in humans, and a large fraction of the selected loci are in canonical non-coding regions that have functional implications as inferred from ENCODE studies.

## Methods

### 1. CHARGE WGS European American samples in this study

The individuals sequenced in this study were part of the Cohorts for Heart and Aging Research in Genetic Epidemiology (CHARGE) cohorts[20], and belong to one of three NHLBI cohort studies. The Atherosclerosis Risk in Communities (ARIC) study[22] contributed 404 participants; the Cardiovascular Health Study (CHS) [23] contributed 237 participants; and the Framingham Heart Study (FHS) [24,25] contributed 321 participants. Each of these cohort studies is briefly described in Suppl. Information.

### 2. Data Generation using whole genome sequencing based on Illumina platforms

Library construction processes for the Illumina pipeline are fully automated at the Baylor College of Medicine Human Genome Sequencing Center (BCM-HGSC). This automated pipeline uses the Biomek NX Span 8 liquid handler in tandem with Biomek FX or NX platforms. Established automated steps within the library construction process include DNA aliquoting, end-repair, 5’ adenylation, adaptor ligation, library amplification and sample pooling using the Biomek Span 8 platform (see Suppl. Information for detailed experimental procedures).

### 3. Alignment, SNP calling and quality assessment

#### 3.1 Read mapping and alignment

The Illumina whole genome sequencing data of CHARGE WGS samples were mapped using BWA[26] against human genome reference sequences (version HG19), and went through sorting, merging, mark-duplicate etc. [4] using the standard Illumina data mapping and BAM finishing pipeline, namely Mercury[27], at BCM-HGSC.

#### 3.2 SNP and genotype calling using SNPTools

An integrative population SNP calling, genotype and phase imputation pipeline named SNPTools[28] were applied to (1) perform SNP sites discovery by considering all samples together, (2) calculate genotype likelihoods at candidate SNP sites for each sample using BAM-specific Binomial Mixture Modeling (BBMM) approach, and (3) refine and impute genotypes calls and phases. We used the default parameters of SNPTools[28] to process CHARGE WGS data, which were tuned in our practice of 1000 Genome project. The software and manual can be downloaded at http://sourceforge.net/projects/snptools/.

#### 3.3 SNP and genotype quality assessments

In total we genotyped 25,135,797 SNPs in 962 CHARGE WGS samples in whole genome, 22.9% are presented in dbSNP (v129). The overall Ti/Tv of all the SNPs is 2.11 and the Non-reference genotype discordance comparing against the SNP array data from 404 ARIC samples is 1.04%. These metrics show the high quality of the SNP and genotype calls.

### 4. Principal Component Analysis

Principal components (PCs) estimated from SNPs of individuals from diverse populations have been shown to correspond with geographic origin^20,21^ and are useful for detecting population structure. Using sequence data from the 962 individuals passing previous quality control, we estimated PCs with the SMARTPCA software[29]. We binned variants into two minor allele frequency (MAF) classes, rare and low frequency variants (MAF 0.5%- 5%), and common variants (MAF >5%). To reduce the impact of linkage disequilibrium (LD) we used PLINK[30] to prune SNPs with a maximum pairwise *r* ^2^ threshold of 0.3, and removed regions with extended LD including the HLA region on chromosome 6. Our final analysis was thus carried out on 1,494,120 rare variants and 513,690 common variants. We then estimated PCs separately from the two variant classes. To evaluate whether the observed structure in CHARGE WGS participants corresponds to individuals of known ancestry, we conducted PCA of genome-wide SNPs in the CHARGE WGS and HGDP[31] participants. We plotted CHARGE WGS participants on PCs estimated from European HGDP populations (Adygei, Basque, Bergamo, French, Orcadian, Russian, Sardinian and Tuscan) and populations from Africa (Mandenka and Yoruba), East Asia (Han Chinese and Japanese), and the Middle East (Druze and Palestinian). Thirty-nine outliers were omitted, resulting in a final study size of 923 participants for subsequent analysis.

### 5. Evidence for capture of the recent and rare variation

Recent sequencing studies have documented a dramatic increase of the effective population size in modern humans[1,3]. One characteristic of this rapid population growth is the elevated number of very rare variants, which are very recent in origin and are enriched for deleterious mutations[32]. Capturing this recent variation is crucial to having an accurate representation of the genetic polymorphism currently segregating in the populations. In order to show that the sequencing depth in the CHARGE WGS data is sufficient to detect very rare variants, we compared the SFS of the CHARGE WGS data to the SFS of both Nelson *et al*. [3] and Tennessen *et al*. [1].

For this purpose, we simulated two populations that follow the same demographic history as in Nelson *et al*.[3] and Tennessen *et al*.[1], and two additional populations following the same models, but without the last epoch of growth. Of importance for demographic consideration, we restricted our analysis to the most homogenous subset of the 923 CHARGE WGS samples, excluding 39 outliers individuals from the PCA (see above). In each simulation, we also use 923 individuals to match the sample size of CHARGE WGS, because the number of rare variants detected (and therefore the shape of the SFS) depends on the sample size[2,33]. We compared the expected SFS of the simulated data with that of the CHARGE WGS data. The results show that SFS of the CHARGE WGS data appears more similar to either published models than to the same models without the final growth epoch. This shows that even with the uncertainty on singletons and very rare variants attributable to the 6.2X coverage of the CHARGE WGS data, the large sample size of the CHARGE WGS data allows one to reasonably capture the recent demographic growth of human populations at a genome-wide level.

### 6. Functional annotation

SNPs were first annotated based on RefSeq[34] using the ANNOVAR program[35]. We used chromatin immunoprecipitation and sequencing (ChIP-seq) data from the ENCODE project[16], and identified putative transcription factor binding sites (TFBSs) using a motif discovery approach (Detailed procedures are presented in Suppl. Information).

### 7. Detecting variants with clinical implications

We identified 1,372 variants in our study as disease-causing (Variant_class is annotated as “DM”) in HGMD database[36], all of which are minor alleles. As expected, most of those mutations are relatively rare (MAF <1%), there are still 120 mutations with MAF = 1–5% and 26 mutations with MAF > 5%. By reviewing the initial literature for the 26 common mutations, we found that 13 were not suggested to be functional from the original reports and 12 were suggested to be functional with partial evidence but without experimental confirmation.

### 8. Natural selection pressure acting on coding and noncoding regions

#### 8.1 Diversity and divergence analysis

To investigate the selection pressure acting on different regions of different genes, we separated gene surrounding regions according to gene functions (biological process terms of Gene Ontology) as well as function domains (1 kb upstream, 5’UTR, exonic+splicing, intronic, 3’UTR and 1kb downstream). We used nucleotide diversity (π)[37] to measure diversity within population and the conservation score GERP++ [38] to measure divergence among species (both are measured per SNP). We divided GERP++ score by its corresponding neutral mutation rate to produce a normalized score (called GERP++ k), for which a smaller number indicates a lower divergence (i.e. higher conservation) on the site. To reduce the large variance in diversity and divergence based on a small number of SNPs, we limited our analysis to major gene/domain group with 100 or more SNPs observed. More details can be found in Suppl. Information 8.

#### 8.2 Detecting signature of natural selection using diversity and divergence analysis in sliding windows

Extremely highly diverse genome regions are candidate targets of diversity-enhancing selection. To identify those regions, a sliding window analysis was conducted on SNPs discovered on 923 European originated individuals based on the PCA analysis. We applied the strict mask for high-mapping-quality from the 1000 Genomes project to the human genome and all regions outside the masked regions were filtered out. Each window is 500 bp wide and the sliding step is 250 bp (i.e. two adjacent windows have 250 bp overlap). We further removed windows with less than half sites (250 bp) masked. Watterson’s θ[39] and nucleotide diversity(π)[37] were calculated for each window using the software jPopGen Suite[40]. The lower bound of recombination events detectable from the haplotypes were calculated using Myers and Griffiths’ algorithm[41] with Liu and Fu’s *R*_*a*_ [42] as the local bound estimation. To speed up the calculation, a maximum of 15 (window size 500 bp) haplotypes were used for each local bound estimation. The number of haplotypes in each window was counted based on SNPtools’ phasing result.

#### 8.3 Using iHS to detect loci that have undergone recent positive selection

In order to perform a positive selection analysis on the CHARGE WGS data, we first removed the 39 individuals identified as outliers in the principal components analysis to reduce population structure confounders. We then removed variants below a 5% minor allele frequency threshold and performed phasing using the program SHAPEIT[43] version 1.532. Using Voight’s iHS method as implemented in the R package REHH, we calculated standardized iHS values genome-wide across the sample[15,44]. Since the iHS method does not provide a formal significance test, we selected the top 1% of the absolute value of the iHS values genome-wide to conduct the analysis with the ENCODE data. To look for regions with a high concentration of iHS hits, we extracted windows of 50 variants that contained more than 11 loci with an absolute value iHS score greater than 2.6, the value of the minimum score of the top 1% of individual iHS hits. We then submitted the top 1% of these windows to the program GREAT[45] for GO analysis. We also compared the distribution of iHS scores between ENCODE functionally annotated categories.

#### 8.4 Purifying selection acting on regulatory regions

We obtained RegulomeDB[46] scores for all non-coding SNPs, with increasing scores suggesting stronger evidence that the SNP may affect gene regulation. We simplified the score categories by combining sub-category into three groups: category 1–2 (very likely affecting binding), category 3–6 (likely affecting some regulatory function) and no-score (likely to be neutral). We separated the SNPs into three groups according to how strong the SNP showing deficiency of homozygote minor allele: genome-wide significant (p-value of Hardy-Weinberg test < 6 x 10^−10^), significant (6 x 10^−10^ ≤ p-value of Hardy-Weinberg test < 1x10^-2^) and the others. We used T-test to compare the difference between the frequencies of SNPs in each of the three RegulomeDB categories between groups.

### 9. Population genomics of non-coding RNAs

A significant proportion of the human genome encodes small and large non-coding RNAs[47] whose patterns of diversity were well captured by these sequence data. To detect signatures of functional constraints on the non-coding RNA regions, we performed population genomic analysis on different classes of non-coding RNAs including microRNA (miRNA), piwi-interacting RNAs (piRNAs) and large intergenic non-coding RNAs (lincRNAs).

#### 9.1 Functional constraints on miRNAs and target sites

The annotations of miRNA precursor and mature miRNA sequences were downloaded from miRBase[48] V19. We defined “conserved” miRNAs by requiring the first 20 nucleotides to be identical between a human mature miRNA and a non-primate mature miRNA as annotated in miRBase[48] V19. We identified 319 human autosomal miRNA precursors that encode conserved miRNAs and the remaining 1160 autosomal miRNA precursor are non-conserved. By polarizing mutations with the *EPO multiple alignments* downloaded from Ensembl database, we found that more than 40% of the derived mutations in the miRNA loci are segregating as singletons in the CHARGE WGS participants.

We investigate the polymorphisms of the conserved miRNAs that are predicted by the TargetScan package[49], including canonical TargetScan based on conservation criteria [49–51] and the Context Score algorithm [50]. We only considered the evolutionarily conserved miRNAs that are incorporated in the TargetScan database which putatively bind 552,104 target sites if we simply apply the “seed matching” rules. We mapped the predicted target sites on the human genome release hg19 using Bowtie [52]. The sites were binned with increasing P_CT_ score (higher P_CT_ score means increasing conservation stringency) or with decreased context scores (lower context score means higher confidence in target prediction).

#### 9.2 Neutral (or nearly) evolutionary patterns of lincRNAs and piRNAs

The genomic coordinates and annotations of lincRNAs, other classes of non-coding RNAs and protein-coding genes were downloaded from the Ensembl (V69) database (www.ensembl.org). The introns and exons of lincRNAs were parsed based on the genomic coordinate information. The frequency spectra analysis on the derived mutations in lincRNAs and piRNAs were based on the *EPO multiple alignments* downloaded from the Ensembl database. We also calculated the genetic diversities of 11,537 pseudogenes annotated in Ensembl database, which putatively serve as a baseline for neutral evolution. To reduce the variation in diversity comparisons, we binned the genome into 10 Mb windows for protein-coding genes, lincRNAs, and pseudogenes; for miRNAs, snoRNAs, snRNAs and piRNAs, we calculated the diversities for each locus.

#### 9.3 GWAS association of mutations in non-coding RNAs and diseases or traits

The SNPs that are significantly associated with human diseases or physiological traits revealed in other studies were taken from GWASdb [53] and Ref [54].

## Results

### Whole genome sequencing of 962 individuals and variant analysis

**Fig 1:**
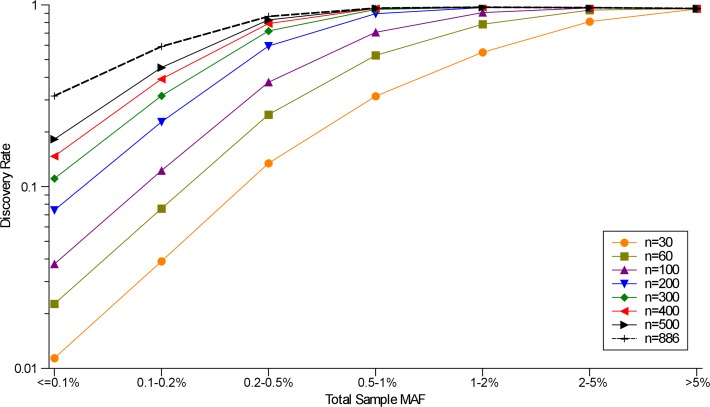
SNP rediscovery rate increases with increasing sample size when using the whole exome sequencing results as the gold standard. The dotted line shows the discovery rates of the total 886 individuals with WGS data. Each point is an average of 100 subsamples of size *n* from 886 individuals. Bootstrap resampling without replacement was carried out for each data point at various sample sizes.

We sequenced the whole genomes of 962 European Americans from the CHARGE consortium[20] at an average depth of 6.2X and applied SNPTools[28] for variant calling. Approximately 25 million SNPs were identified across the 22 autosomes (with an overall transition/transversion ratio of 2.13, **Table A in S1 File**). Of these, 64.3% were of low frequency [minor allele frequency (MAF) <1%] and a large proportion of these (49.2%) were unique to this study. By comparing SNPs identified in 886 overlapping samples having high coverage whole-exome capture sequence (WECS) (average coverage = 115X per sample) data, the overall genotype (including heterozygotes and homozygotes) concordance rate was higher than 99% across the allele frequency spectrum (**Table A in S1 File**, **Fig. A in S1 File**). The rediscovery probability in these data for sites with MAF>0.5% and a sample size of ≥500 is greater than 95% when compared to the deep coverage WECS data (**Fig. 1**). When MAF = 0.2–0.5%, the rediscovery probability remained as high as 80% (**Fig. 1**).

Comparison of the site frequency spectrum (SFS) with recently published data[1,3] shows that the SFS of the CHARGE data is similar to recently published models that include a recent epoch of rapid growth[1,3], documenting that the large sample size and sequencing depth give us adequate power to capture the dramatic inflation in singletons triggered by such recent and rapid growth[33] (**Fig. B in S1 File**). Furthermore, our large sample will provide improved power to detect SNPs having potential functional impact. We identified 1,372 variants in our study as disease-causing in the HGMD database[36] (**S1 Table**), all of which are the minor alleles. On average, each individual carries 21.37 (*sd* = 4.47) putatively disease-causing alleles (**Fig. C in S1 File**). Principal component analysis (PCA) suggested population substructure within the study participants, including a group of 31 outliers (**Fig. D in S1 File**) which are likely of partially Middle Eastern ancestry **(Fig. E in S1 File)**. Eight additional individuals of East Asian ancestry **(Fig. F in S1 File)** were also omitted from further analysis, resulting in 923 individuals that were used for subsequent analysis of demographic history and natural selection.

### Population genetic analysis flags loci with signatures of natural selection

**Fig 2:**
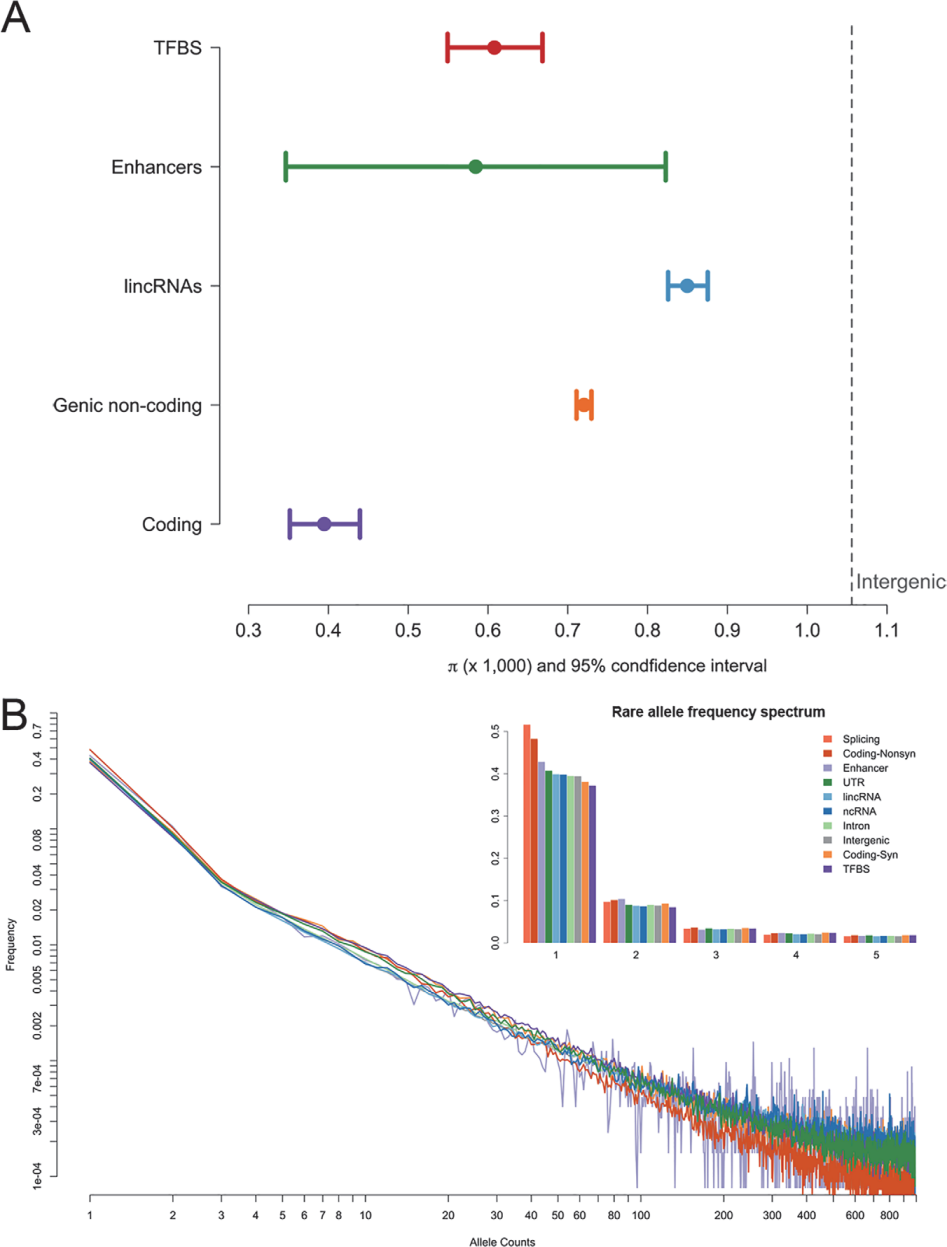
Genetic diversity across functional categories in the CHARGE-S participants. **A** is the distribution of nucleotide diversity across different categories of non-coding regions. **B** presents the frequency spectra of the minor allele in the studied population. The *x*-axis is the number of minor alleles in each non-coding category and the *y*-axis is the frequency of the chromosomes that carry that allele.

The relatively large sample size allowed us to explore patterns of genetic variation, including low frequency variants, and to contrast those patterns across gene families and across annotation categories. The HLA gene cluster demonstrated the highest nucleotide diversity, measured by Watterson’s θ [39], the number of observed haplotypes, minimum number of recombination events for the haplotypes[41,42], and nucleotide diversity (π)[37] (**Fig. G in S1 File**). The distribution of nucleotide diversity stratified by functional annotations (see Methods and Suppl. Information 6) demonstrated clear heterogeneity across functional categories (**Fig. 2A**). Consistent with other studies of diversity in samples of European ancestry[5], coding regions have the lowest level of π (mean = 0.39 x 10^−3^) among functional categories, and intergenic regions have the highest (mean = 1.06 x 10^−3^). Transcription factor binding sites (TFBSs) and enhancers have similar mean π values of 0.61 x 10^−3^ and 0.58 x 10^−3^, respectively. Splicing and non-synonymous SNPs show the strongest enrichment of rare variants, with splicing mutations having the highest proportion of singletons, and a relatively low proportion of high frequency variants (**Fig. 2B**), consistent with strong purifying selection[1,5]. We observe no significant differences between the SFSs of non-coding SNPs with different regulatory potential as annotated by ENCODE[46]. On the other hand, we observed a significant enrichment of likely regulatory variants from a subset of SNPs that largely deviate from Hardy-Weinberg equilibrium (**Fig. H in S1 File**). These observations suggest that the majority of the SNPs located within potential protein binding regions are likely neutral, while a small proportion are functional and under purifying selection. This may be one small piece of evidence that the claim that 80% of the genome is functionally constrained is an over-estimate.

**Fig 3:**
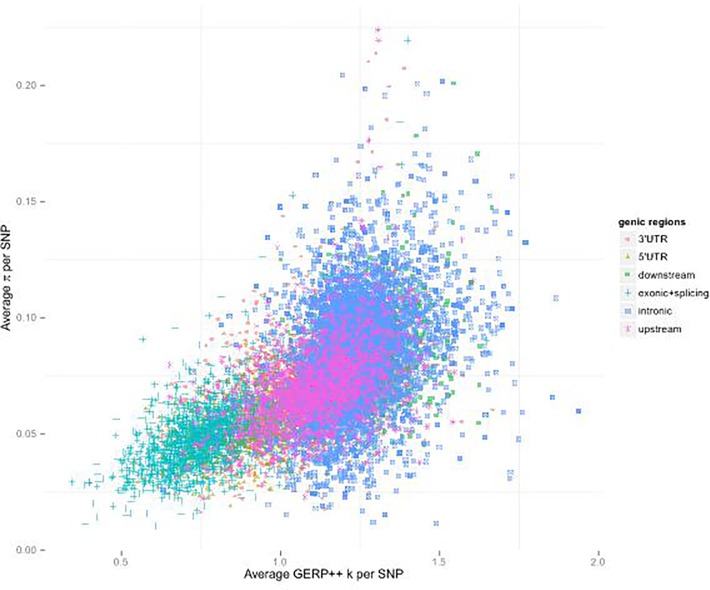
The average π and GERP++ k of the SNPs discovered in 14,501 major domains of genic regions x function groups. π measures nucleotide pairwise difference and the conservation score GERP++ measures divergence among species. GERP++ score is divided by its corresponding neutral mutation rate to produce a normalized score (called GERP++ k), for which a smaller number indicates a lower divergence (i.e. higher conservation) on the site.

By analyzing the different measures of diversity, evidence of multiple forms of natural selection was detected across SNPs grouped according to predicted functional domains two-way classified by genic regions (exonic, intronic, 5’ UTR, 3’ UTR, upstream and downstream) and gene function groups (Gene Ontology) (see **Fig. 3** and **S1 File)**. Exonic regions as well as 5’ UTRs, 3’ UTRs and upstream regions demonstrated low diversity (measured by per SNP π) and low divergence (measured by per SNP GERP++ k score), consistent with purifying selection acting on functional important regions. The distributions of 5’ UTRs, 3’ UTRs and upstream regions have substantial overlaps with exonic regions, suggesting the functional importance of some of those noncoding regions. Among the 14,501 major functional domains (with 100 or more SNPs observed), many with both low nucleotide diversity and low divergence across species have functions related to early development, especially neural system development, or housekeeping functions (**Table B in S1 File**). Functional domains showing both high diversity and high divergence are significantly enriched in immune response (**Table C in S1 File**), which might be shaped by balancing selection or diversity-enhancing selection. It is notable that 3’ UTRs of genes related to positive regulation of metalloenzyme activity, “positive regulation of transferase activity” and “embryo development ending in birth or egg hatching” also show high nucleotide diversity and high divergence.

**Fig 4:**
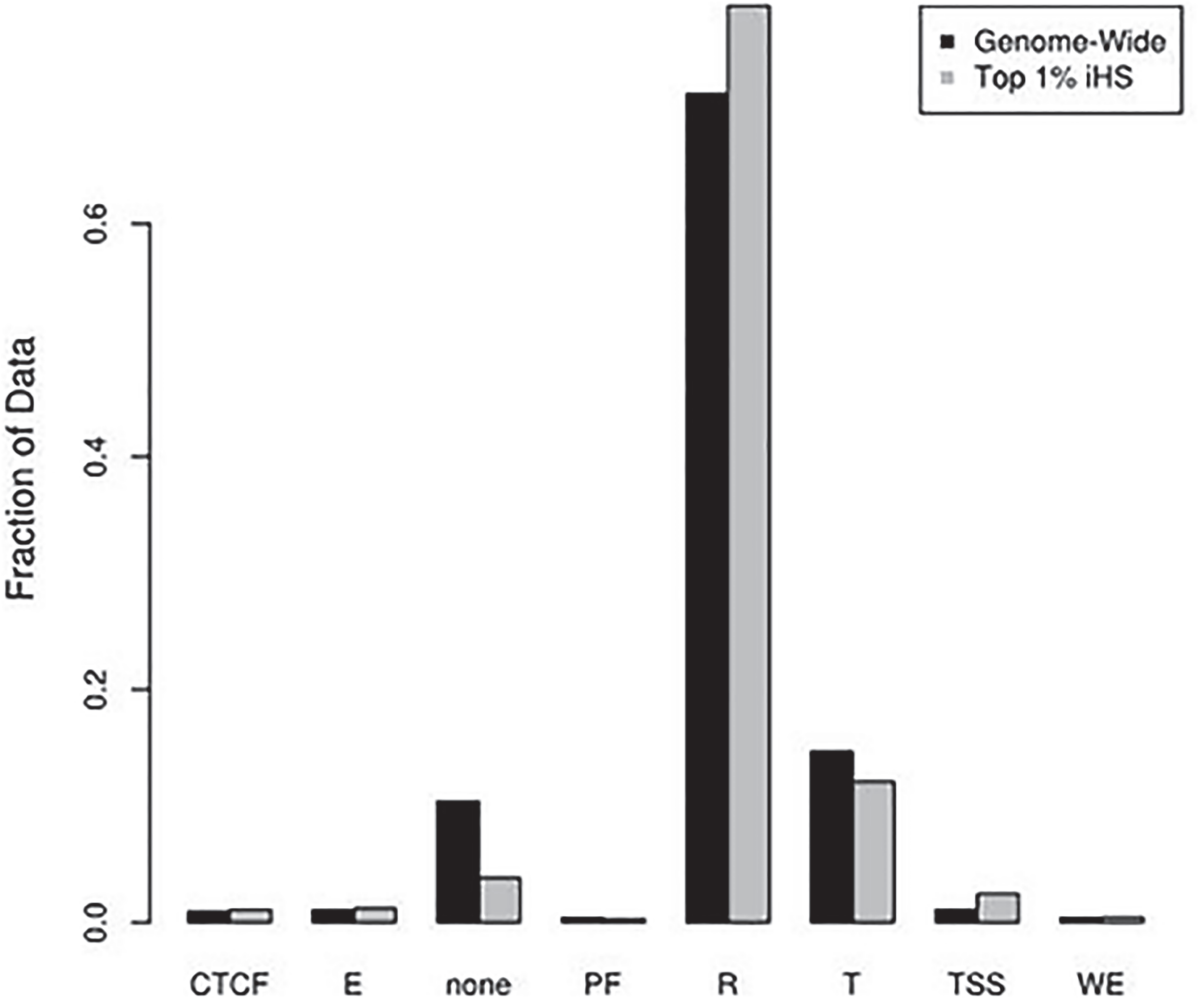
Distribution of ENCODE functional regions genome-wide and in the top 1% of iHS hits. Categories are the 7 identified ENCODE functional regions inferred from the combined ChromHMM and Segway results plus the “none” category indicating loci without functional annotation. All categories except for weak enhancers (WE) show significant differences between the two datasets with a *P* <0.0063 by a χ^2^ test. CTCF: CTCF enriched element; WE:Weak Enhancer; T:Transcribed Region; E: Enhancer; PF:Promoter Flank; R: Repressed/Low Activity; TSS: promoter region including transcription start site.

Haplotype-based tests provide excellent power to detect positive selection occurring in human populations within the last 25,000 years in both coding and non-coding regions[55]. We conducted a positive selection scan using the haplotype-based method iHS[15] and identified many loci putatively under positive selection in the sample. The results show that approximately 60% of the top 1% of selection signals lie in intergenic regions, 33% in intronic regions, and slightly over 1% in genic regions. Using the seven functionally annotated regions inferred from the ENCODE project[16], we determined the distribution of the ENCODE regions in the top 1% of iHS hits and compared this distribution to the respective genome-wide span of each annotation. Loci in predicted repressed or low activity regions (R) and loci near or overlapping the transcription start site (TSS) were the most significantly over-represented in the top 1% of iHS hits when compared to the genome-wide distribution (**Fig. 4**). Loci in transcribed (T) regions or loci with no ENCODE functional annotation were the most significantly under-represented in the top 1% of iHS scores. Only the weak enhancers (WE) showed no significant difference with the genome-wide distribution. These results appear to indicate that a great deal of recent selection is acting on non-coding functional elements within the genome.

### Selection signals are pervasive in non-coding regions

**Fig 5:**
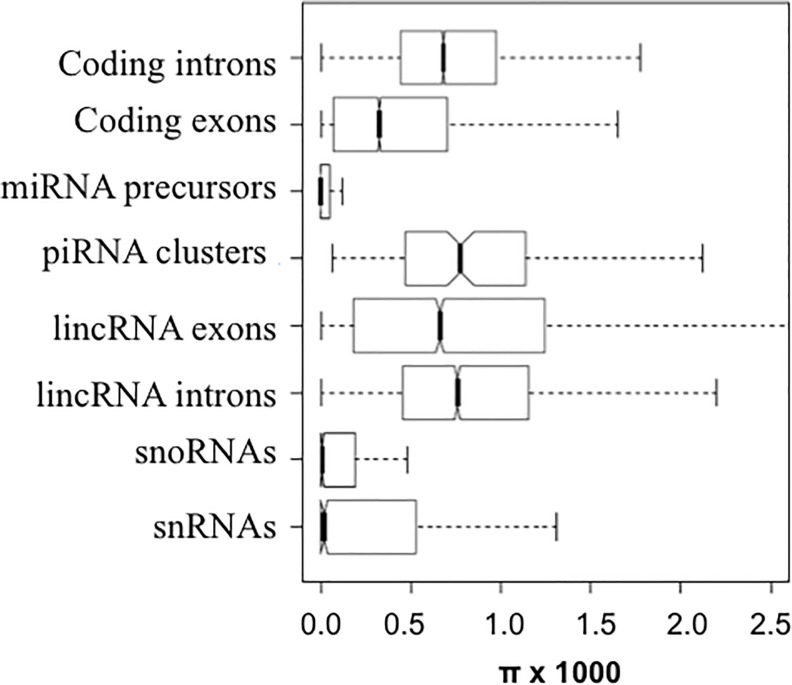
Boxplots of the nucleotide diversity (π) in different classes of non-coding RNAs. For comparison, the diversities in pseudogenes, coding sequences, and introns of protein-coding regions are also plotted.

**Fig 6:**
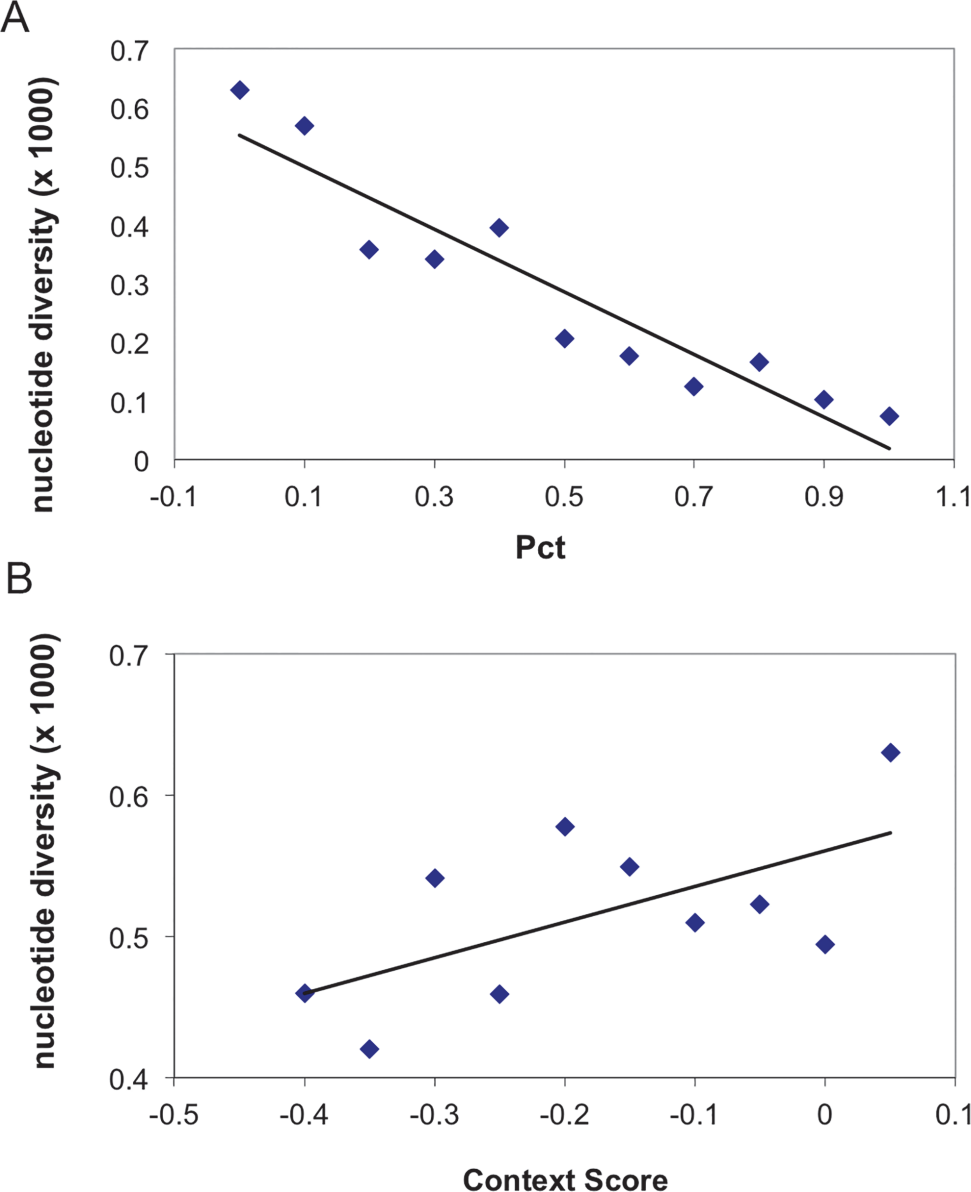
Nucleotide diversities (π) in the 3’UTRs and miRNA target sites that are identified in the TargetScan program. **A** the target sites of the conserved miRNAs that are identified with conservation criteria of miRNA-target pairing. Sites are classified with increasing P_CT_ score, which means higher stringency criteria. P_CT_ = 0 means the nucleotide sites in the 3’ UTRs are not inside any “seed pairing” regions. **B** the target sites of the conserved miRNAs that are identified with the context score of the miRNA pairing. Smaller context scores mean the target sites have high probability to be regulated by miRNAs. The nucleotide sites in the 3’ UTRs that are not inside any “seed pairing” regions have a context score of 0.05.

Overall there was strong evidence for heterogeneity of evolutionary forces that act on different classes of non-coding RNAs. A significant proportion of the human genome encodes small and large non-coding RNAs[47] whose patterns of diversity were well captured by sequence data from a large sample size from a single population. miRNAs are small non-coding RNAs that modulate the expression level of target transcripts by targeting 3’ UTRs[56]. Among the 1,479 miRNA loci currently annotated in human autosomes, we identified 1,106 SNPs. However with many SNPs in non-conserved miRNAs, 50% of the miRNA loci do not harbor any mutations, yielding an median π near 0, significantly lower than the value obtained over pseudogenes [π ± sd is (0.906 ± 0.366) × 10^−3^; *P* < 10^−16^, **Fig. 5**]. These mutations are significantly over-represented in lowly expressed miRNAs and under-represented in highly expressed miRNAs, since highly expressed miRNAs are generally evolutionarily conserved[57] (**Table D in S1 File**). Also, nucleotide diversities are generally lower in the 3’ UTRs [π is (0.648 ± 1.043)×10^−3^] than in pseudogenes (*P* <10^−10^, Kolmogorov-Smirnov test). We observed significant reduction in polymorphism level in miRNA target sites that are identified either with conservation criteria (P_CT_) or without conservation criteria (the context score algorithm) in TargetScan predictions[58] (**Fig. 6**). Not surprisingly, π in the target sites decreases as the stringency of conservation increases (Pearson’s correlation coefficient *r* = -0.92, *P* < 0.0001, **Fig. 6A**). Interestingly, diversity is also significantly reduced in the target sites that are predicted with the context score algorithm (without conservation criteria), although we observed a marginal correlation between π and context score (Spearman’s correlation coefficient is 0.49, *P* = 0.15, **Fig. 6B**). SFS analysis indicates most derived mutations in miRNA loci (**Fig. I in S1 File**) and miRNA target sites (**Fig. J in S1 File,** either predicted with conservation criteria or context score) are under strong purifying selection, since those derived mutations are significantly skewed towards low frequencies. Overall there is a pattern of strong purifying selection acting on the mutations in miRNA and their binding sites. However, we also identified a handful of mutations in mature miRNAs that are segregating at intermediate or high frequencies in these CHARGE participants (**Table E in S1 File**), suggesting further studies are needed to examine roles of these mutations in human health and reproductive fitness. Three mutations in miRNA precursors that are segregating at high frequencies in these samples are overlapping with SNPs that are significantly associated with “metabolite levels” or “lung adenocarcinoma” as identified in previous GWAS studies (**S2 Table**).

We also detected signatures of functional constraints on snoRNAs and snRNAs (**Fig. 5**). However, our analysis indicates that mutations in lincRNAs and piRNAs are generally under neutral or weak selective pressure (Suppl. Information). The gene structures of lincRNAs (long intergenic noncoding RNAs) are similar to protein-coding genes in terms of exons and introns, nevertheless, they lack the capacity to encode proteins[59]. Among the 5,610 autosomal lincRNAs annotated by Ensembl (R69), π in the exons and introns of the lincRNAs are very similar [π ± sd is (0.872 ± 0.300)×10^−3^ and (0.878 ± 0.278)×10^−3^ in exons and introns, respectively]. It is notable that π in both introns and exons of lincRNAs are generally not different from pseudogenes (P > 0.05 in both cases, **Fig. 5**), suggesting they are generally under neutral or very weak functional constraint. piRNAs (piwi-interacting RNAs) are small non-coding RNAs transcribed from large clusters in the germline cells[60]. For the ~200 piRNA clusters identified in human genome[61], π ± sd is (0.885 ± 0.579)×10^−3^, a level of nucleotide diversity similar to lincRNAs (**Fig. 5**). Although the rapid evolution of some lincRNAs and piRNAs might be driven by positive selection, analysis of frequency spectra of derived mutations suggests that both classes of ncRNAs overall are evolving neutrally or under weak functional constraint (**Fig. K in S1 File**).

Previous studies revealed that sequences of lincRNAs can evolve rapidly while their function is still conserved[62,63]. Here we ask whether the lincRNAs and piRNAs which overall show neutral evolutionary patterns have an impact on human phenotypes or diseases. Strikingly, we found ~670 mutations in lincRNAs captured in the CHARGE-S participants to be overlapping with SNPs associated with human disease or other medical traits in published GWAS studies (**S2 Table**). Numerous mutations in piRNA loci also overlap with SNPs that are associated human phenotypes. It is notable that about 90 mutations in a piRNA cluster (chr4: 10114931–10157431) that is upstream of *SLC2A9* gene have high derived allele frequencies in the CHARGE participants, and these mutations are significantly enriched for SNPs associated with serum uric acid levels[64] (**S2 Table**). Since serum uric acid levels are associated with increased risk of heart disease and other physiological phenotypes[64], our result suggests further investigations are needed to study function of this cluster of piRNAs.

## Conclusion

The whole-genome sequencing effort in this study for the first time has allowed us to probe the role of natural selection in large samples of individuals from a single population. Our analysis finds that protein-coding genes provide only a small fraction of the targets of selection, and pervasive selection has operated on functional non-coding genomic regions. The selection signals in both directions (positive and negative) that are outliers from distributions of population genetics statistics are orthogonal to other prioritization methods for genome regions such as the sequence conservation or functional annotations/predictions. Our study indicates that applying rigorous population genetics tests holds promise to provide a more complete and accurate picture for of the evolutionary forces that act on functional elements in the non-coding regions.

## Supporting Information

S1 File Contains Fig. A, Heterozygous concordance when comparing SNPs from WGS and WECS data. Fig. B, Site Frequency Spectrum (SFS) of the CHARGE WGS data compared to published demographic models. Fig. C, Distribution of the number of disease-causing alleles an individual carries in 962 CHARGE WGS participants. Fig. D, Principal components of genetic variation in CHARGE WGS participants estimated from (a) common variants (minor allele frequency > 5%) and (b) rare variants (minor allele frequency between 0.5–5%). Fig. E, Principal components of genetic variation in HGDP participants with European or Middle Eastern ancestry with CHARGE WGS participants projected onto the PCs. Fig. F, Principal components of genetic variation in HGDP participants with European or East Asian ancestry with CHARGE WGS participants projected onto the PCs. Fig. G, Four diversity measures of 500 bp sliding windows and iHS scores across 22 autosomes. Fig. H, Enrichment of SNPs residing in regulatory regions in the group of SNPs showing deficiency of homozygote of minor allele. Fig. I, The distributions of derived allele frequencies (DAF) in miRNA precursor, mature miRNA and seed regions. Fig. J, Distributions of derived allele frequencies (DAF) in 3’ UTRs and miRNA target sites. Fig. K, Distributions of derived allele frequencies (DAF) in introns and exons of lincRNAs, piRNAs and introns of coding regions. Table A, SNP calling quality summary. Table B, Top 20 domains with both low diversity and low divergence. Table C, Top 20 domains with both high diversity and high divergence. Table D, Highly expressed miRNAs are generally conserved across species and have lower diversity in CHARGE WGS participants. Table E, 42 mutations re-captured in this study are located in mature miRNAs and are segregating at intermediate to high frequencies (derived allele frequency >5% in the CHARGE WGS participants).
(DOC)

S1 Table Putative disease-causing mutations in the 962 European individuals when annotating using Human Genetics Mutation Database (HGMD). (XLS)

S2 Table CHARGE WGS mutations in non-coding RNAs overlapped with GWAS hits with the associated diseases or traits. (XLS)
