# Supplementary material for: Population Genomic Analysis of 962 Whole Genome Sequences of Humans Reveals Natural Selection in Non-Coding Regions
Source: PLoS One. 2015 Mar 25;10(3):e0121644. doi: 10.1371/journal.pone.0121644 (PMC4373932; doi:10.1371/journal.pone.0121644)

# Supplementary Information

Table of Contents

1. CHARGE WGS European American samples in this study [4](#__RefHeading___Toc286412746)

Atherosclerosis Risk in Communities (ARIC) study [4](#__RefHeading___Toc286412747)

Cardiovascular Health Study (CHS) [4](#__RefHeading___Toc286412748)

Framingham Heart Study (FHS) [4](#__RefHeading___Toc286412749)

2. Data Generation using whole genome sequencing based on Illumina platforms [4](#__RefHeading___Toc286412750)

3. Alignment, SNP calling and quality assessment [5](#__RefHeading___Toc286412751)

Read mapping and alignment [5](#__RefHeading___Toc286412752)

SNP and genotype calling using SNPTools [5](#__RefHeading___Toc286412753)

SNP and genotype quality assessments [5](#__RefHeading___Toc286412754)

4. Principal component analysis [6](#__RefHeading___Toc286412755)

5. Evidence for capture of the recent and rare variation [6](#__RefHeading___Toc286412756)

6. Functional annotation [7](#__RefHeading___Toc286412757)

7. Detecting variants with clinical implications [8](#__RefHeading___Toc286412758)

8. Natural selection pressure acting on coding and noncoding regions [9](#__RefHeading___Toc286412759)

Diversity and divergence analysis [9](#__RefHeading___Toc286412760)

Detecting signature of natural selection using diversity and divergence analysis in sliding windows [9](#__RefHeading___Toc286412761)

Using iHS to detect loci that have undergone recent positive selection [10](#__RefHeading___Toc286412762)

Purifying selection acting on regulatory regions [10](#__RefHeading___Toc286412763)

9. Population genomics of non-coding RNAs [11](#__RefHeading___Toc286412764)

Functional constraints on miRNAs and target sites [11](#__RefHeading___Toc286412765)

Neutral (or nearly) evolutionary patterns of lincRNAs and piRNAs [12](#__RefHeading___Toc286412766)

GWAS association of mutations in non-coding RNAs and diseases or traits [12](#__RefHeading___Toc286412767)

References [14](#__RefHeading___Toc286412768)

Supplementary Tables [18](#__RefHeading___Toc286412769)

Table A. SNP calling quality summary [18](#__RefHeading___Toc286412770)

Table B. Top 20 domains with both low diversity and low divergence. [19](#__RefHeading___Toc286412771)

Table C. Top 20 domains with both high diversity and high divergence. [20](#__RefHeading___Toc286412772)

Table D. Highly expressed miRNAs are generally conserved across species and have lower diversity in CHARGE WGS participants [21](#__RefHeading___Toc286412773)

Table E. 42 mutations re-captured in this study are located in mature miRNAs and are segregating at intermediate to high frequencies (derived allele frequency >5% in the CHARGE WGS participants). [22](#__RefHeading___Toc286412774)

Supplementary Figures [23](#__RefHeading___Toc286412775)

Figure A. Heterozygous concordance when comparing SNPs from WGS and WECS data. [23](#__RefHeading___Toc286412776)

Figure B. Site Frequency Spectrum (SFS) of the CHARGE WGS data compared to published demographic models. [24](#__RefHeading___Toc286412777)

Figure C. Distribution of the number of disease-causing alleles an individual carries in 962 CHARGE WGS participants. [25](#__RefHeading___Toc286412778)

Figure D. Principal components of genetic variation in CHARGE WGS participants estimated from (a) common variants (minor allele frequency > 5%) and (b) rare variants (minor allele frequency between 0.5-5%). [26](#__RefHeading___Toc286412779)

Figure E. Principal components of genetic variation in HGDP participants with European or Middle Eastern ancestry with CHARGE WGS participants projected onto the PCs. [28](#__RefHeading___Toc286412780)

Figure F. Principal components of genetic variation in HGDP participants with European or East Asian ancestry with CHARGE WGS participants projected onto the PCs. [29](#__RefHeading___Toc286412781)

Figure G. Four diversity measures of 500 bp sliding windows and iHS scores across 22 autosomes. [30](#__RefHeading___Toc286412782)

Figure H. Enrichment of SNPs residing in regulatory regions in the group of SNPs showing deficiency of homozygote of minor allele. [31](#__RefHeading___Toc286412783)

Figure I. The distributions of derived allele frequencies (DAF) in miRNA precursor, mature miRNA and seed regions. [32](#__RefHeading___Toc286412784)

Figure J. Distributions of derived allele frequencies (DAF) in 3’ UTRs and miRNA target sites. [33](#__RefHeading___Toc286412785)

Figure K. Distributions of derived allele frequencies (DAF) in introns and exons of lincRNAs, piRNAs and introns of coding regions. [35](#__RefHeading___Toc286412786)

# 1. CHARGE WGS European American samples in this study

The individuals sequenced in this study were part of the Cohorts for Heart and Aging Research in Genetic Epidemiology (CHARGE) cohorts, and belong to one of three NHLBI cohort studies. The ARIC study contributed 404 participants; the Cardiovascular Health Study contributed 237 participants; and the Framingham Heart Study contributed 321 participants. Each of these cohort studies is briefly described below.

## Atherosclerosis Risk in Communities (ARIC) study

The ARIC study has been described in detail previously. White and African American men and women aged 45-64 years at baseline were recruited from four communities: Forsyth County, North Carolina; Jackson, Mississippi; Minneapolis, Minnesota; and Washington County, Maryland. A total of 15,792 individuals participated in the baseline examination in 1987-1989, with three triennial follow-up examinations.

## Cardiovascular Health Study (CHS)

CHS has been described in detail previously. CHS is a population-based cohort study of risk factors for coronary heart disease and stroke in adults ≥65 years conducted across four field centers. The original cohort of 5,201 persons was recruited in 1989-1990 from random samples of the Medicare eligibility lists.

## Framingham Heart Study (FHS)

FHS has been described in detail previously. Individuals were initially recruited in 1948 in Framingham, MA to evaluate cardiovascular disease risk factors. The second generation cohort (5,124 offspring of the original cohort) was recruited between 1971 and 1975, and multiple lipid measurements where available were averaged. The third generation cohort (4,095 grand-children of the original cohort) was collected between 2002 and 2005, and a single lipid measurement was available.

# 2. Data Generation using whole genome sequencing based on Illumina platforms

Library construction processes for the Illumina pipeline are fully automated at the Baylor College of Medicine Human Genome Sequencing Center (BCM-HGSC). This automated pipeline uses the Biomek NX Span 8 liquid handler in tandem with Biomek FX or NX platforms. Established automated steps within the library construction process include DNA aliquoting, end-repair, 5’ adenylation, adaptor ligation, library amplification and sample pooling using the Biomek Span 8 platform. SPRI-bead purification associated with end-repair, nick translation and PCR amplification steps are all performed on Biomek FX and NX platforms. All processes within library construction are fully LIMS integrated for tracking sample identities on reaction plates to prevent sample swaps. LIMS interfacing allows downstream sequencing data to be deconvoluted, with reads being assigned to the appropriate barcodes/libraries/samples during analysis. To date, over 15,000 libraries have been completed using these automated methods for capture and WGS applications with up to 96 sample barcodes now employed for multiplexing. For this project, Illumina PE libraries were barcoded with standard Illumina multiplex adaptors and pooled for sequencing in sets of three samples to generate an average of 6.2-fold sequence coverage per sample.

Methods for WGS sequencing followed standard Illumina PairEnd library protocols with minor modifications. DNA concentration was determined by pico green assays while DNA integrity was determined through Agilent Bioanalyzer traces and agarose gels. Libraries were constructed using 1ug of genomic DNA in 100ul volume and sheared into fragments of approximately 300 base pairs in a Covaris plate with E210 system (Covaris, Inc. Woburn, MA). The setting was 10% Duty cycle, Intensity of 4, 200 Cycles per Burst, for 120 seconds. Fragment size was checked using a 2.2 % Flash Gel DNA Cassette (Lonza, Cat. No.57023). The fragmented DNA was end-repaired in 90 μl total reaction volume containing sheared DNA, 9 μl 10X buffer, 5 μl END Repair Enzyme Mix and H_2_O (NEBNext End-Repair Module; Cat. No. E6050L) and then incubated at 20°C for 30 min. A-tailing was performed in a total reaction volume of 60 μl containing end-repaired DNA, 6 μl 10X buffer, 3 μl Klenow Fragment (NEBNext dA-Tailing Module; Cat. No. E6053L) and H_2_O followed by an incubation at 37°C for 30 min. Illumina multiplex adapter ligation (NEBNext Quick Ligation Module Cat. No. E6056L) was performed in a total reaction volume of 90 μl containing 18 μl 5X buffer, 5 μl ligase, 0.5 μl 100 μM adaptor and H_2_O at room temperature for 30 min. After ligation, PCR with Illumina PE 1.0 and modified barcode primers was performed in 170 μl reactions containing 85 2x Phusion High-Fidelity PCR master mix, adaptor ligated DNA, 1.75 μl of 50 μM each primer and H_2_O. The standard thermocycling for PCR was 5’ at 95°C for the initial denaturation followed by 6-10 cycles of 15 s at 95°C, 15 s at 60°C and 30 s at 72°C and a final extension for 5 min at 72°C. Agencourt® XP® Beads (Beckman Coulter Genomics, Inc.; Cat. No. A63882) was used to purify DNA after each enzymatic reaction. After bead purification, PCR product quantification and size distribution was determined using the Caliper GX 1K/12K/High Sensitivity Assay Labchip (Hopkinton, MA, Cat. No. 760517). Mean depth of coverage was 6.2x, with minimum coverage of 4.0x and maximum coverage of 17.4x.

# 3. Alignment, SNP calling and quality assessment

## Read mapping and alignment

The Illumina whole genome sequencing data of CHARGE WGS samples were mapped using BWA (v0.5.9-r16) against human genome reference sequences (version HG19), and went through sorting, merging, mark-duplicate etc. using the standard Illumina data mapping and BAM finishing pipeline, namely Mercury, at BCM-HGSC.

## SNP and genotype calling using SNPTools

An integrative population SNP calling, genotype and phase imputation pipeline named SNPTools were applied to (1) perform SNP sites discovery by considering all samples together, (2) calculate genotype likelihoods at candidate SNP sites for each sample using BAM-specific Binomial Mixture Modeling (BBMM) approach, and (3) refine and impute genotypes calls and phases.

We used the default parameters of SNPTools to process CHARGE WGS data, which were tuned in our practice of 1000 Genome project. The software and manual can be downloaded at this link (<http://sourceforge.net/projects/snptools/>).

## SNP and genotype quality assessments

The statistics and quality assessments of the final SNP and genotype calls results are summarized in **Table A**. In total we genotyped 25,135,797 SNPs in 962 CHARGE WGS samples in whole genome, 22.9% are presented in dbSNP (v129). The overall Ti/Tv of all the SNPs is 2.11 and the Non-reference genotype discordance comparing against the SNP array data from 404 ARIC samples is 1.04%. These metrics show the high quality of the SNP and genotype calls.

The overall allele frequency spectrum is concordant with expectations from such a large sample: 40% of the SNPs are singletons, 30% are rare (allele frequency < 5% excluding singleton) and 30.3% of the SNPs are common. Comparing to dbSNP(v129), 64.5% of common SNPs are known, while for singleton and rare SNPs, only 9.1% and 1.6% are known.

While sample size did not affect genotype concordance rate, SNPs with higher MAF in general have higher heterozygote concordance rate, with the lowest rate of 80% for MAF=0.1-0.2%. Singletons (MAF < 0.1%) had exceptionally high concordance rate (99%) (**Figure A**), due to much more stringent cutoffs applied.

# 4. Principal component analysis

Principal components (PCs) estimated from SNPs of individuals from diverse populations have been shown to correspond with geographic origin[^20^](#_ENREF_20),[^21^](#_ENREF_21) and are useful for detecting population structure. Using sequence data from the 962 individuals passing previous quality control, we estimated PCs with the SMARTPCA software. We binned variants into two minor allele frequency (MAF) classes, rare and low frequency variants (MAF 0.5%- 5%), and common variants (MAF >5%). To reduce the impact of linkage disequilibrium (LD) we used PLINK to prune SNPs with a maximum pairwise *r*^2^ threshold of 0.3, and removed regions with extended LD including the HLA region on chromosome 6. Our final analysis was thus carried out on 1,494,120 rare variants and 513,690 common variants. We then estimated PCs separately from the two variant classes.

In both variant classes, the first PC appears as a gradient with a distinct cluster of 31 individuals (**Figure D**). PC2 appears to capture different information from common and rare variants. In the common variant PCA, the second PC distinguishes individuals of the ARIC and FHS cohorts (**Figure Da)**, while in the rare variant PCA this distinction is less clear with individuals from all three cohorts located at the negative extreme of PC2 (**Figure Db**). To evaluate whether the difference was due to the greater number of SNPs in the rare variant PCA, we evaluated PCs from a randomly thinned set of 598,158 rare variants. PCs from thinned and unthinned rare variants were qualitatively similar (data not shown), indicating that the difference in common and rare variant PCA is not attributable to the number of SNPs in the analysis.

To evaluate whether the observed structure in CHARGE WGS participants corresponds to individuals of known ancestry we conducted PCA of genome-wide SNPs in the CHARGE WGS and HGDP participants. We downloaded HGDP data from the CEPH foundation website (http://www.cephb.fr/en/hgdp/), consisting of Affymetrix Human Mapping 500k array SNP data for 5 individuals per population. Chromosome and genomic position were lifted from hg build 18 to 19 based on the UCSC liftover chain file and dbSNP build 135. CHARGE WGS variants were subsetted to include only SNPs on the Affymetrix 500k array from chromosome and physical position information. We used PLINK to prune SNPs in LD with maximum pairwise *r*^2^ of 0.3 for a final set of 131,894 SNPs. Due to the ascertainment of SNPs on the Affymetrix 500k array, the final set of SNPs consists primarily of common SNPs, i.e., ~82% of SNPs have MAF > 0.05 in the CHARGE WGS participants.

We plotted CHARGE WGS participants on PCs estimated from European HGDP populations (Adygei, Basque, Bergamo, French, Orcadian, Russian, Sardinian and Tuscan) and populations from Africa (Mandenka and Yoruba), East Asia (Han Chinese and Japanese), and the Middle East (Druze and Palestinian). The 31 previously identified CHARGE WGS outlier individuals appear to share recent common ancestry with Middle Eastern reference populations (**Figure E**). Eight additional individuals appear to share recent common ancestry with East Asian reference populations (**Figure F**). These 39 outliers were omitted, resulting in a final study size of 923 participants for subsequent analysis.

## 5. Evidence for capture of the recent and rare variation

Recent sequencing studies have documented a dramatic increase of the effective population size in modern humans. One characteristic of this rapid population growth is the elevated number of very rare variants, which are very recent in origin and are enriched for deleterious mutations. Capturing this recent variation is crucial to having an accurate representation of the genetic polymorphism currently segregating in the populations.

Discovery of very rare variants is better achieved with large sample size and deep coverage. In order to show that the sequencing depth in the CHARGE WGS data is sufficient to detect very rare variants, we compared the SFS of the CHARGE WGS data to the SFS of both Nelson *et al.* and Tennessen *et al.* .

For this purpose, we simulated two populations that follow the same demographic history as in Nelson *et al.* and Tennessen *et al.*, and two additional populations following the same models, but without the last epoch of growth. Of importance for demographic consideration, we restricted our analysis to the most homogenous subset of the 923 CHARGE WGS samples, excluding 39 outliers individuals from the PCA (see above). In each simulation, we also use 923 individuals to match the sample size of CHARGE WGS, because the number of rare variants detected (and therefore the shape of the SFS) depends on the sample size. We compared the expected SFS of the simulated data with that of the CHARGE WGS data.

The results show that SFS of the CHARGE WGS data **(Figure B)** appears more similar to either published models than to the same models without the final growth epoch. This shows that even with the uncertainty on singletons and very rare variants attributable to the 6.2X coverage of the CHARGE WGS data, the large sample size of the CHARGE WGS data allows one to reasonably capture the recent demographic growth of human populations at a genome-wide level.

It is important to note that despite the sample size adjustment, the three SFS are not expected to be exactly identical. The variations in the SFS do not directly reflect differences in power to detect rare variation, since they are influenced by many other factors. For example, Nelson *et al.* and Tennessen *et al.* involved different individuals, have different power (different original sample size, population homogeneity), used different statistical approaches to model demography, and therefore produced different demographic models of European history. In addition, CHARGE WGS includes data from the whole genome while the other two studies are based on the exome only. The non-exome part of the genome is expected to be on average under less selection, and therefore present a slightly lower proportion of extremely rare variants.

# 6. Functional annotation

SNPs were first annotated based on RefSeq using the ANNOVAR program. Ancestral allele of each SNP was determined according to the ancestral reference sequence produced by the 1000 Genomes Project (www.1000genomes.org). In addition to the gene-based annotations, we aimed to annotate non-genic SNPs within known functional regions, in order to learn more about the patterns of genetic variation in these domains. We used chromatin immunoprecipitation and sequencing (ChIP-seq) data from the ENCODE project, and identified putative transcription factor binding sites (TFBSs) using a motif discovery approach. Motif discovery and binding site identification were performed using ChIP-seq peak data released by the ENCODE project and available from the UCSC genome browser version hg19 (genome.ucsc.edu). All peaks in both the HAIB and SYDH datasets, unrestricted prior to July 2012, and excluding controls, time-course experiments, or those obtained after chemical stimulation, were downloaded.

Motif discovery was run using the MEME program. First, four subsamples of 1000 ChIP-seq peaks for each experiment and replicate (each corresponding to a given transcription factor and cell-type) were obtained and trimmed to 100 bp, either at the center of the peak region (HAIB) or centered on the position of the peak called within the region (SYDH). MEME was then run on the corresponding collection of human reference sequences for each subsample, directly and after masking repetitive sequences with RepeatMasker, for a total of eight runs per experiment and replicate. In each case, the program was configured to search for the three best scoring motifs (-nmotifs 3) between 6 and 25 bp long (-minw 6 -maxw 25), to consider both the forward and reverse strands (-revcomp), and to allow zero or one occurrence of the motif per sequence (-mod zoops).

The motifs returned by MEME for each of the eight runs in each case were then manually inspected, compared, and verified against the JASPAR 2009 (http://jaspar.genereg.net/) and UNIPROBE databases (http://the_brain.bwh.harvard.edu/uniprobe/). Any experiment where no high quality consensus motif was identified across sets, replicates, or showing disagreements for transcription factors (TFs) found in both the HAIB and SYDH datasets were discarded. The best motif for each TF was then selected to run the MAST program from the MEME Suite for binding site identification over the full set of peak regions for each ChIP-seq experiment. Only hits with a *P*-value < 0.0001 and E-value < 10, both corrected for sequence composition, were retained. For the HAIB dataset, where peak regions were produced separately for each experimental replicate, transcription factor binding sites (TFBS) not found in both replicates were discarded. At this point, degenerate positions were eliminated from the edges of all TFBS and motifs, by trimming those with an information content < 0.5 in the motif away from each side. The union of all TFBS identified for each TF, merging across both, HAIB and SYDH datasets, and cell types, were kept for further analysis. Finally, any TF with less than 500 TFBS were removed, leaving a total of 78 TFs and 2,013,074 TFBS with an average of 25,808 binding sites per TF. In addition, we included information on non-coding developmental enhancers that were experimentally shown to drive expression in the mouse embryo, as well as long intergenic non-coding RNAs (lincRNAs), which are covered by 13,376 and 1,991,089 SNPs, respectively.

# 7. Detecting variants with clinical implications

Since our sample size is relatively large for a single population sample, we have improved power to detect SNPs with large functional impact. We identified 1,372 variants in our study as disease-causing (Variant_class is annotated as “DM”) in HGMD database (**S1 Table**), all of which are minor alleles. As expected, most of those mutations are relatively rare (MAF <1%), there are still 120 mutations with MAF = 1-5% and 26 mutations with MAF > 5%. On average each individual carries 21 putative disease causing alleles (minimum 10, maximum 40) (**Figure C**). This seems to be a huge mutation burden on individual genome. By reviewing the initial literature for the 26 common mutations (**S1 Table**), we found that 13 were not suggested to be functional from the original reports and 12 were suggested to be functional with partial evidence but without experimental confirmation or whose risks were suggested to be depended on other mutations. The remaining SNP (rs6092) is a non-synonymous mutation reported to cause plasminogen activator inhibitor 1 deficiency, a rare autosomal recessive hematologic disorder. As the allele has a frequency of 12% in our samples and among them 14 are homozygotes, the mutation alone should not be sufficient to produce a clinical phenotype. Another example is a mutation in the 3’ UTR region of IGF-1 gene (rs70961704). It has been reported to result in deregulated IGF-1 mRNA maturation and cause insulin-like growth factor deficiency, a rare autosomal recessive disease. The causative allele has a frequency of more than 3%. Such high frequency is not consistent with the rareness of the disease. Although we did not fully investigate all mutations, what we found from those “common” disease-causing mutations suggests that a large proportion of the mutations reported causing Mendelian type diseases may be only partially responsible for the diseases, whose penetrance depends on epistasis of other phenotype modifiers, either genetic or environmental.

# 8. Natural selection pressure acting on coding and noncoding regions

## Diversity and divergence analysis

Diversity (within a population) and divergence (between species) measures are powerful indicators of natural selection acting on DNA polymorphism. To investigate the selection pressure acting on different regions of different genes, we separated gene surrounding regions according to gene functions (biological process terms of Gene Ontology) as well as function domains (1 kb upstream, 5’UTR, exonic+splicing, intronic, 3’UTR and 1kb downstream). We used nucleotide diversity (π) to measure diversity within population and the conservation score GERP++ to measure divergence among species (both are measured per SNP, not per region/domain). We divided GERP++ score by its corresponding neutral mutation rate to produce a normalized score (called GERP++ k), for which a smaller number indicates a lower divergence (i.e. higher conservation) on the site. To reduce the large variance in diversity and divergence based on a small number of SNPs, we limited our analysis to major gene/domain group with 100 or more SNPs observed. **Figure 3** shows the average π and GERP++ k per SNP discovered in 14,501 major domains of gene groups. Many domains with both low diversity and low divergence have functions related to development, especially neural system development, and housekeeping functions (**Table B showing the top 20 domains**). On the other hand, many domains with both high diversity and high divergence have functions related to immune response (**Table C showing the top 20 domains**), which is likely shaped by balancing selection.

## Detecting signature of natural selection using diversity and divergence analysis in sliding windows

Extremely highly diverse genome regions are candidate targets of diversity-enhancing selection. To identify those regions, a sliding window analysis was conducted on SNPs discovered on 923 European originated individuals based on the PCA analysis. We applied the strict mask for high-mapping-quality from the 1000 Genomes project to the human genome and all regions outside the masked regions were filtered out. Each window is 500 bp wide and the sliding step is 250 bp (i.e. two adjacent windows have 250 bp overlap). We further removed windows with less than half sites (250 bp) masked. Watterson’s θ and nucleotide diversity(π) were calculated for each window using the software jPopGen Suite. In short, $\theta=S/a_{n}$, where *S* is the total number of polymorphic sites in a sample of *n* sequences and $a_{n}=\sum_{i=1}^{n-1} \frac{1}{i}$; $\Pi=\frac{2}{n(n-1)}\sum_{i<j} d_{ij}$, where *d_ij_* is the number of nucleotide differences between sequences *i* and *j*. The lower bound of recombination events detectable from the haplotypes were calculated using Myers and Griffiths’ algorithm with Liu and Fu’s  *R_a_* as the local bound estimation. To speed up the calculation, a maximum of 15 (window size 500 bp) haplotypes were used for each local bound estimation. The number of haplotypes in each window was counted based on SNPtools’ phasing result.

The diversity circos figure of the four measure along with iHS scores (see below) is shown in **Figure G**. By far the most diverse region on the genome is located on chromosome 6 where HLA genes cluster, as indicated by high peaks of multiple diversity measures and iHS score. Another observation of the landscapes is that the peak patterns are often coordinated in different diversity measures. We defined a window as extreme diverse if at least three out of four of its measures are 3 standard deviations larger than the means. A total of 9,297 extreme diverse windows were identified. The majority (5,779) are in intergenic regions. The remaining windows are in vicinity of ~1000 coding genes or non-coding RNAs, among which 3,274 are in intronic regions, 138 are in 1 kb upstream or downstream regions and 106 are in exonic regions of coding genes or non-coding RNAs.

Many of the known candidate genes under balancing or positive selection have exonic or upstream/downstream windows been identified as extreme diverse, including *HLA-B, HLA-C, HLA-DPA1, HLA-DPB1, HLA-DQA1, HLA-DQA2, HLA-DQB1, HLA-DQB2, ABO, ALPK2, BLK, BTNL2/HCG23, CD6, DEFB1, IL17RC, MICA, PAMR1, PKD1L2, PSORS1C1/CDSN, OR52E2, OR5P2, OVCH2, OVGP1, SLC14A1, WWOX*. Most of those windows are outside coding regions, suggesting selections acting on regulatory regions. For example, the window chr18:43302763-43303263 locates at the upstream of *SLC14A1*, an established candidate gene under balancing selection, and overlaps with its TFBS region according to ENCODE. Another example is the intron 5 of *FHIT*. SNPs in this intron are associated with the risk of prostate cancer, and the intron has been suspected under balancing or positive selection in human and non-human primates, and having regulatory function. We identified 12 extreme diverse windows within introns of this gene and most of them are located in intron 5, confirming this intron may be a target of balancing or positive selection. The gene with the largest number (673) of extreme diverse windows in vicinity is *CSMD1* (**Figure G**). It is a regulator of the complement system, whose function includes response to viruses and inflammatory reactions, and its SNPs have been shown to be significant correlated with virus diversity, which makes it a good candidate gene under diversity-enhancing selection. Interestingly, all extremely diverse windows of this gene are in introns, which suggests that the introns may have regulatory functions or be under some diversity-enhancing selection.

## Using iHS to detect loci that have undergone recent positive selection

In order to perform a positive selection analysis on the CHARGE WGS data, we first removed the 39 individuals identified as outliers in the principal components analysis to reduce population structure confounders. We then removed variants below a 5% minor allele frequency threshold and performed phasing using the program SHAPEIT version 1.532. Using Voight’s iHS method as implemented in the R package REHH, we calculated standardized iHS values genome-wide across the sample. Since the iHS method does not provide a formal significance test, we selected the top 1% of the absolute value of the iHS values genome-wide to conduct the analysis with the ENCODE data.

To look for regions with a high concentration of iHS hits, we extracted windows of 50 variants that contained more than 11 loci with an absolute value iHS score greater than 2.6, the value of the minimum score of the top 1% of individual iHS hits. We then submitted the top 1% of these windows to the program GREAT for GO analysis. The GO analysis reveals biological processes including detection of mechanical stimulus involved in sensory perception of sound. One example of highly concentrated iHS scores occurred in the gene *espin* (*ESPN*) on chromosome 1. This gene is involved in sensory transduction in mechanical and chemical stimuli and is associated with several forms of deafness.

## Purifying selection acting on regulatory regions

We obtained RegulomeDB scores for all non-coding SNPs, with increasing scores suggesting stronger evidence that the SNP may affect gene regulation. We simplified the score categories by combining sub-category into three groups: category 1-2 (very likely affecting binding), category 3-6 (likely affecting some regulatory function) and no-score (likely to be neutral). Consistent with previous studies, we observed little difference among the SNPs of the three binned categories as to the proportions of rare (MAF<1%), low-frequency (MAF 1-5%) and common (MAF>5%) SNPs (data not shown). We further grouped the SNPs according to how strong the SNP showing deficiency of homozygote minor allele: genome-wide significant (p-value of Hardy-Weinberg test < 6 x 10^-10^), significant (6 x 10^-10^ ≤ p-value of Hardy-Weinberg test < 1x10^-2^) and the others. We observed significant enrichment of category 1-2 SNPs when comparing genome-wide significant group to the others group (p-value < 5 x 10^-6^, T test, one tail) and when comparing significant group to the others group (p-value < 6 x 10^-10^, T test, one tail). The enrichment of category 1-2 and 3-6 combined is even more significant, with both p-values < 1 x 10^-16^ when comparing the two significant groups to the others group (**Figure H**). These observations suggest that the majority of the SNPs located within potential protein binding regions are likely neutral while a small proportion are functional and under purifying selection.

# 9. Population genomics of non-coding RNAs

A significant proportion of the human genome encodes small and large non-coding RNAs whose patterns of diversity were well captured by these sequence data. To detect signatures of functional constraints on the non-coding RNA regions, we performed population genomic analysis on different classes of non-coding RNAs including microRNA (miRNA), piwi-interacting RNAs (piRNAs) and large intergenic non-coding RNAs (lincRNAs).

## Functional constraints on miRNAs and target sites

The annotations of miRNA precursor and mature miRNA sequences were downloaded from miRBase V19. Among the 1,479 miRNA loci that are annotated in the human autosomes, with our sequencing results, we identified 1,106 SNPs (after quality filtering) in the CHARGE participants in these miRNA precursors (41.2% of these mutations are singletons and 9.6% of them are doubletons). Functional miRNAs are usually evolutionarily conserved and highly expressed while the non-conserved miRNAs are either evolutionarily transient or driven by positive Darwinian selection . We defined “conserved” miRNAs by requiring the first 20 nucleotides to be identical between a human mature miRNA and a non-primate mature miRNA as annotated in miRBase V19. With this criterion, we identified 319 human autosomal miRNA precursors that encode conserved miRNAs and the remaining 1160 autosomal miRNA precursor are non-conserved. Based on the miRNA deep sequencing results compiled in miRBase V19, we classified autosomal miRNAs into three categories: highly expressed, with total NGS (next generation sequencing) reads from all experiments ≥ 500; medium expressed, with NGS reads between 20 and 500; and lowly expressed, with NGS reads less than 20. In total we identified 253, 377 and 849 highly-, median- and lowly-expressed miRNA loci, respectively. A salient observation is that the highly expressed miRNAs are highly significantly enriched in the evolutionary conserved class and the lowly expressed miRNAs are significantly enriched in non-conserved class (*P* < 10^-16^, χ^2^ test, **Table D**). Not surprisingly, π is significantly lower in the highly expressed miRNA precursors than in the median (*P* = 0.009, Kolmogorov-Smirnov test) or than in the lowly expressed miRNA precursors (*P* <10^-4^, Kolmogorov-Smirnov test, **Table D**). Previous studies indicate mature miRNAs are generally under stronger selective constraints than other regions of miRNA precursors and we also identified reduced π in mature miRNAs comparing the whole miRNA precursors (**Table D**).

By polarizing mutations with the EPO multiple alignments downloaded from Ensembl database, we found that more than 40% of the derived mutations in the miRNA loci are segregating as singletons in the CHARGE WGS participants (**Figure Ia**). The analysis of frequency spectra of the derived alleles indicates mutations in mature miRNAs or seed regions tend to be more skewed to low frequencies than other mutations in the miRNA precursors, consistent with the nucleotide diversity comparisons (**Table D**). The pattern is more striking for the miRNA loci that are conserved between human and other non-primate species (**Figure Ib**). We identified 330 mutations in total located in mature miRNAs and 144 of these (44%) are segregating as singletons in the CHARGE WGS participants. We identified 62 derived mutations in mature miRNAs that are segregating at > 5% in the surveyed populations (20 of them are located in miRNAs derived from transposable elements and 42 mutations are remaining if we exclude these transposable-element-derived miRNAs, **Table E**), suggesting further studies are needed to examine roles of these mutations in human health and environmental adaptation.

Previous studies have indicated that mutations in miRNA target sites appear to be under strong purifying selection and those mutations will significantly affect the expression patterns of the target genes by re-wiring the regulatory networks . Herein we investigate the polymorphisms of the conserved miRNAs that are predicted by the TargetScan package, including canonical TargetScan based on conservation criteria and the Context Score algorithm . We only considered the evolutionarily conserved miRNAs that are incorporated in the TargetScan database which putatively bind 552,104 target sites if we simply apply the “seed matching” rules. We mapped the predicted target sites on the human genome release hg19 using Bowtie . The sites were binned with increasing P_CT_ score (higher P_CT_ score means increasing conservation stringency) or with decreased context scores (lower context score means higher confidence in target prediction). The nucleotide diversity analysis indicated reduction in polymorphism in miRNA target sites than in 3’ UTRs regions that do not harbor seed-pairing sites (**Figure 6**). The frequency spectra analysis indicate most derived mutations in the miRNA target sites (either predicted with conservation criteria or context score) are under strong purifying selection since those derived mutations are significantly skewed towards low frequencies (**Figure Ja and b**). In summary, our population genetic analysis indicates that strong purifying selection has acted on the mutations in the miRNA regulatory networks.

## Neutral (or nearly) evolutionary patterns of lincRNAs and piRNAs

We did not identify signatures of functional constrains on long intergenic non-coding RNAs (lincRNAs) and very weak signature of selective pressure on piwi-interacting RNAs (piRNAs). lincRNAs are a large class of long intergenic RNAs that affect the expression patterns of the target genes, and they span about 129 Mb in human genomes. The gene structure of lincRNAs are similar to protein-coding genes in terms of exons and introns, nevertheless, they lack coding capacity.  The genomic coordinates and annotations of lincRNAs, other classes of non-coding RNAs and protein-coding genes were downloaded from the Ensembl (V69) database ([www.ensembl.org](http://www.ensembl.org/)). The introns and exons of lincRNAs were parsed based on the genomic coordinate information. Among the 1463 snoRNAs and 1821 snRNAs annotated in Ensembl (V69), at least 45% of these two classes of non-coding rRNAs have θ_π_ values smaller than 1.0×10^-5^. Genomic annotations of ~200 piRNA clusters identified in human testes were taken from and re-mapped on hg19. The frequency spectra analysis on the derived mutations in lincRNAs and piRNAs were based on the EPO multiple alignments downloaded from the Ensembl database (**Figure K**). We also calculated the genetic diversities of 11,537 pseudogenes annotated in Ensembl database, which putatively serve as a baseline for neutral evolution. To reduce the variation in diversity comparisons, we binned the genome into 10 Mb windows for protein-coding genes, lincRNAs, and pseudogenes; for miRNAs, snoRNAs, snRNAs and piRNAs, we calculated the diversities for each locus.

## GWAS association of mutations in non-coding RNAs and diseases or traits

The SNPs that are significantly associated with human diseases or physiological traits revealed in other studies were taken from GWASdb and Ref . The mutations captured in this study that are overlapping with previously identified GWAS-SNPs are presented in **S2 Table.**

# References

1. Psaty BM, O'Donnell CJ, Gudnason V, Lunetta KL, Folsom AR, et al. (2009) Cohorts for Heart and Aging Research in Genomic Epidemiology (CHARGE) Consortium: Design of prospective meta-analyses of genome-wide association studies from 5 cohorts. Circ Cardiovasc Genet 2: 73-80.

2. ARIC I (1989) The Atherosclerosis Risk in Communities (ARIC) Study: design and objectives. American Journal of Epidemiology 129: 687-702.

3. Fried L, Borhani N, Enright P, Furberg C, Gardin J, et al. (1991) The Cardiovascular Health Study: design and rationale. Annals of Epidemiology 1: 263-276.

4. Dawber T, Meadors G, Moore F (1951) Epidemiological approaches to heart disease: the Framingham Study. Am J Public Health Nations Health 41: 279-281.

5. Feinleib M, Kannel W, Garrison R, McNamara P, Castelli W (1975) The Framingham Offspring Study. Design and preliminary data. Prevention Medicine 4: 518-525.

6. Li H, Durbin R (2009) Fast and accurate short read alignment with Burrows-Wheeler transform. Bioinformatics 25: 1754-1760.

7. Marth GT, Yu F, Indap AR, Garimella K, Gravel S, et al. (2011) The functional spectrum of low-frequency coding variation. Genome Biol 12: R84.

8. Reid J, Carroll A, Veeraraghavan N, Dahdouli M, Sundquist A, et al. (2014) Launching genomics into the cloud: deployment of Mercury, a next generation sequence analysis pipeline. BMC Bioinformatics 15: 30.

9. Wang Y, Lu J, Yu J, Gibbs RA, Yu F (2013) An integrative variant analysis pipeline for accurate genotype/haplotype inference in population NGS data. Genome Research.

10. Patterson N, Price AL, Reich D (2006) Population structure and eigenanalysis. Plos Genetics 2: 2074-2093.

11. Purcell S, Neale B, Todd-Brown K, Thomas L, Ferreira MA, et al. (2007) PLINK: a tool set for whole-genome association and population-based linkage analyses. Am J Hum Genet 81: 559-575.

12. Cann HM, de Toma C, Cazes L, Legrand MF, Morel V, et al. (2002) A human genome diversity cell line panel. Science 296: 261-262.

13. Herraez DL, Bauchet M, Tang K, Theunert C, Pugach I, et al. (2009) Genetic Variation and Recent Positive Selection in Worldwide Human Populations: Evidence from Nearly 1 Million SNPs. Plos One 4.

14. Nelson MR, Wegmann D, Ehm MG, Kessner D, St Jean P, et al. (2012) An abundance of rare functional variants in 202 drug target genes sequenced in 14,002 people. Science 337: 100-104.

15. Tennessen JA, Bigham AW, O'Connor TD, Fu W, Kenny EE, et al. (2012) Evolution and functional impact of rare coding variation from deep sequencing of human exomes. Science 337: 64-69.

16. Fu W, O'Connor TD, Jun G, Kang HM, Abecasis G, et al. (2013) Analysis of 6,515 exomes reveals the recent origin of most human protein-coding variants. Nature 493: 216-220.

17. Coventry A, Bull-Otterson LM, Liu X, Clark AG, Maxwell TJ, et al. (2010) Deep resequencing reveals excess rare recent variants consistent with explosive population growth. Nat Commun 1: 131.

18. Keinan A, Clark AG (2012) Recent explosive human population growth has resulted in an excess of rare genetic variants. Science 336: 740-743.

19. Pruitt KD, Tatusova T, Brown GR, Maglott DR (2012) NCBI Reference Sequences (RefSeq): current status, new features and genome annotation policy. Nucleic Acids Res 40: D130-135.

20. Wang K, Li MY, Hakonarson H (2010) ANNOVAR: functional annotation of genetic variants from high-throughput sequencing data. Nucleic Acids Research 38.

21. Consortium EP, Dunham I, Kundaje A, Aldred SF, Collins PJ, et al. (2012) An integrated encyclopedia of DNA elements in the human genome. Nature 489: 57-74.

22. Bailey TL, Elkan C (1994) Fitting a mixture model by expectation maximization to discover motifs in biopolymers. Proc Int Conf Intell Syst Mol Biol 2: 28-36.

23. Smit A, Hubley R, Green P. RepeatMasker Open-3.0. 1996-2010

<http://www.repeatmasker.org>

24. Bailey TL, Boden M, Buske FA, Frith M, Grant CE, et al. (2009) MEME SUITE: tools for motif discovery and searching. Nucleic Acids Res 37: W202-208.

25. Visel A, Minovitsky S, Dubchak I, Pennacchio LA (2007) VISTA Enhancer Browser - a database of tissue-specific human enhancers. Nucleic Acids Research 35: D88-D92.

26. Stenson PD, Ball EV, Howells K, Phillips AD, Mort M, et al. (2009) The Human Gene Mutation Database: providing a comprehensive central mutation database for molecular diagnostics and personalized genomics. Hum Genomics 4: 69-72.

27. Bonapace G, Concolino D, Formicola S, Strisciuglio P (2003) A novel mutation in a patient with insulin-like growth factor 1 (IGF1) deficiency. J Med Genet 40: 913-917.

28. Oleksyk TK, Smith MW, O'Brien SJ (2010) Genome-wide scans for footprints of natural selection. Philos Trans R Soc Lond B Biol Sci 365: 185-205.

29. Nei M, Li WH (1979) Mathematical model for studying genetic variation in terms of restriction endonucleases. Proc Natl Acad Sci U S A 76: 5269-5273.

30. Davydov EV, Goode DL, Sirota M, Cooper GM, Sidow A, et al. (2010) Identifying a high fraction of the human genome to be under selective constraint using GERP++. PLoS Comput Biol 6: e1001025.

31. Watterson GA (1975) On the number of segregating sites in genetical models without recombination. Theor Popul Biol 7: 256-276.

32. Liu X (2012) jPopGen Suite: population genetic analysis of DNA polymorphism from nucleotide sequences with errors. Methods in Ecology and Evolution 3: 624-627.

33. Myers SR, Griffiths RC (2003) Bounds on the minimum number of recombination events in a sample history. Genetics 163: 375-394.

34. Liu X, Fu YX (2008) Algorithms to estimate the lower bounds of recombination with or without recurrent mutations. BMC Genomics 9 Suppl 1: S24.

35. Krzywinski M, Schein J, Birol I, Connors J, Gascoyne R, et al. (2009) Circos: an information aesthetic for comparative genomics. Genome Res 19: 1639-1645.

36. Fumagalli M, Cagliani R, Pozzoli U, Riva S, Comi GP, et al. (2009) Widespread balancing selection and pathogen-driven selection at blood group antigen genes. Genome Research 19: 199-212.

37. Ding MC, Wang Q, Lo EH, Stanley GB (2011) Cortical Excitation and Inhibition following Focal Traumatic Brain Injury. Journal of Neuroscience 31: 14085-14094.

38. Fumagalli M, Pozzoli U, Cagliani R, Comi GP, Bresolin N, et al. (2010) Genome-Wide Identification of Susceptibility Alleles for Viral Infections through a Population Genetics Approach. Plos Genetics 6.

39. Delaneau O, Marchini J, Zagury JF (2012) A linear complexity phasing method for thousands of genomes. Nat Methods 9: 179-181.

40. Voight BF, Kudaravalli S, Wen X, Pritchard JK (2006) A map of recent positive selection in the human genome. PLoS Biol 4: e72.

41. Gautier M, Vitalis R (2012) rehh: an R package to detect footprints of selection in genome-wide SNP data from haplotype structure. Bioinformatics 28: 1176-1177.

42. McLean CY, Bristor D, Hiller M, Clarke SL, Schaar BT, et al. (2010) GREAT improves functional interpretation of cis-regulatory regions. Nat Biotechnol 28: 495-501.

43. Naz S, Griffith AJ, Riazuddin S, Hampton LL, Battey JF, Jr., et al. (2004) Mutations of ESPN cause autosomal recessive deafness and vestibular dysfunction. J Med Genet 41: 591-595.

44. Boyle AP, Hong EL, Hariharan M, Cheng Y, Schaub MA, et al. (2012) Annotation of functional variation in personal genomes using RegulomeDB. Genome Res 22: 1790-1797.

45. Esteller M (2011) Non-coding RNAs in human disease. Nat Rev Genet 12: 861-874.

46. Kozomara A, Griffiths-Jones S (2011) miRBase: integrating microRNA annotation and deep-sequencing data. Nucleic Acids Res 39: D152-157.

47. Bartel DP (2004) MicroRNAs: genomics, biogenesis, mechanism and function. Cell 116: 281-297.

48. Lu J, Shen Y, Wu Q, Kumar S, He B, et al. (2008) The birth and death of microRNA genes in Drosophila. Nat Genet 40: 351-355.

49. Zhang R, Peng Y, Wang W, Su B (2007) Rapid evolution of an X-linked microRNA cluster in primates. Genome Res 17: 612-617.

50. Lu J, Fu Y, Kumar S, Shen Y, Zeng K, et al. (2008) Adaptive evolution of newly emerged micro-RNA genes in Drosophila. Mol Biol Evol 25: 929-938.

51. Lu J, Clark AG (2012) Impact of microRNA regulation on variation in human gene expression. Genome Res.

52. Saunders MA, Liang H, Li W-H (2007) Human polymorphism at microRNAs and microRNA target sites. Proceedings of the National Academy of Sciences 104: 3300-3305.

53. Friedman RC, Farh KK, Burge CB, Bartel DP (2009) Most mammalian mRNAs are conserved targets of microRNAs. Genome Res 19: 92-105.

54. Grimson A, Farh KK, Johnston WK, Garrett-Engele P, Lim LP, et al. (2007) MicroRNA targeting specificity in mammals: determinants beyond seed pairing. Mol Cell 27: 91-105.

55. Lewis BP, Burge CB, Bartel DP (2005) Conserved seed pairing, often flanked by adenosines, indicates that thousands of human genes are microRNA targets. Cell 120: 15-20.

56. Langmead B, Trapnell C, Pop M, Salzberg SL (2009) Ultrafast and memory-efficient alignment of short DNA sequences to the human genome. Genome Biol 10: R25.

57. Guttman M, Amit I, Garber M, French C, Lin MF, et al. (2009) Chromatin signature reveals over a thousand highly conserved large non-coding RNAs in mammals. Nature 458: 223-227.

58. Girard A, Sachidanandam R, Hannon GJ, Carmell MA (2006) A germline-specific class of small RNAs binds mammalian Piwi proteins. Nature 442: 199-202.

59. Li MJ, Wang P, Liu X, Lim EL, Wang Z, et al. (2012) GWASdb: a database for human genetic variants identified by genome-wide association studies. Nucleic Acids Res 40: D1047-1054.

60. Johnson AD, O'Donnell CJ (2009) An open access database of genome-wide association results. BMC Med Genet 10: 6.

# Supplementary Tables

### Table A. SNP calling quality summary

|  | #SNP | Ti/TV | dbSNP% (V129) |
| --- | --- | --- | --- |
| # Total SNPs | 25135797 | 2.11 | 22.9% |
| Singleton | 9915657 | 1.99 | 1.6% |
| Rare (MAF ≤ 5%) | 7606990 | 2.39 | 9.1% |
| Common (>5%) | 7613150 | 2.02 | 64.5% |

### Table B. Top 20 domains with both low diversity and low divergence.

| ***GO term (gene group)*** | ***Nvar*** | ***ave_k*** | ***se_k*** | ***ave_pi*** | ***se_pi*** | ***Domain*** |
| --- | --- | --- | --- | --- | --- | --- |
| **post-embryonic camera-type eye development** | 119 | 0.39 | 0.052 | 0.011 | 0.0036 | exonic+splicing |
| **regulation of keratinocyte proliferation** | 102 | 0.42 | 0.061 | 0.014 | 0.0057 | exonic+splicing |
| **noradrenergic neuron differentiation** | 103 | 0.41 | 0.060 | 0.020 | 0.0034 | exonic+splicing |
| **neuroligin clustering** | 100 | 0.34 | 0.053 | 0.024 | 0.0074 | exonic+splicing |
| **organ formation** | 140 | 0.48 | 0.051 | 0.019 | 0.0040 | exonic+splicing |
| **gephyrin clustering** | 155 | 0.38 | 0.049 | 0.028 | 0.0068 | exonic+splicing |
| **peripheral nervous system neuron development** | 147 | 0.34 | 0.049 | 0.029 | 0.0067 | exonic+splicing |
| **lung saccule development** | 121 | 0.49 | 0.063 | 0.018 | 0.0031 | exonic+splicing |
| **paraxial mesodermal cell fate commitment** | 107 | 0.42 | 0.058 | 0.027 | 0.0050 | exonic+splicing |
| **postsynaptic membrane assembly** | 163 | 0.40 | 0.050 | 0.029 | 0.0067 | exonic+splicing |
| **postsynaptic density protein 95 clustering** | 163 | 0.40 | 0.050 | 0.029 | 0.0067 | exonic+splicing |
| **chromatin-mediated maintenance of transcription** | 221 | 0.48 | 0.051 | 0.022 | 0.0042 | exonic+splicing |
| **negative regulation of pathway-restricted SMAD protein phosphorylation** | 118 | 0.52 | 0.071 | 0.015 | 0.0038 | exonic+splicing |
| **striatal medium spiny neuron differentiation** | 139 | 0.47 | 0.058 | 0.025 | 0.0058 | exonic+splicing |
| **negative regulation of proteasomal ubiquitin-dependent protein catabolic process** | 114 | 0.52 | 0.068 | 0.018 | 0.0068 | exonic+splicing |
| **regulation of actin filament polymerization** | 102 | 0.43 | 0.066 | 0.029 | 0.0085 | exonic+splicing |
| **alternative nuclear mRNA splicing; via spliceosome** | 133 | 0.53 | 0.057 | 0.016 | 0.0045 | exonic+splicing |
| **neural tube formation** | 171 | 0.49 | 0.058 | 0.026 | 0.0060 | exonic+splicing |
| **nucleosome disassembly** | 223 | 0.50 | 0.052 | 0.024 | 0.0048 | exonic+splicing |
| **positive regulation of transporter activity** | 114 | 0.53 | 0.065 | 0.018 | 0.0059 | exonic+splicing |

Note: nvar: number of SNPs in the domain; ave_k: average of GERP++ k per SNP; se_k: standard error of ave_k; ave_pi: average of nucleotide diversity per SNP; se_pi: standard error of ave_pi.

### Table C. Top 20 domains with both high diversity and high divergence.

| ***GO term (gene group)*** | ***nvar*** | ***ave_k*** | ***se_k*** | ***ave_pi*** | ***se_pi*** | ***Domain*** |
| --- | --- | --- | --- | --- | --- | --- |
| **antigen processing and presentation of exogenous peptide antigen via MHC class I, TAP-independent** | 307 | 1.40 | 0.062 | 0.22 | 0.0099 | Exonic +splicing |
| **antigen processing and presentation of exogenous peptide antigen via MHC class I, TAP-independent** | 234 | 1.31 | 0.067 | 0.22 | 0.012 | Upstream |
| **detection of bacterium** | 211 | 1.31 | 0.066 | 0.22 | 0.013 | Upstream |
| **antigen processing and presentation of exogenous peptide antigen via MHC class I, TAP-independent** | 204 | 1.55 | 0.077 | 0.20 | 0.011 | Downstream |
| **regulation of interleukin-4 production** | 344 | 1.51 | 0.054 | 0.20 | 0.0096 | Intronic |
| **regulation of interleukin-10 secretion** | 344 | 1.51 | 0.054 | 0.20 | 0.0096 | Intronic |
| **positive regulation of metalloenzyme activity** | 116 | 1.30 | 0.098 | 0.21 | 0.020 | 3'UTR |
| **positive regulation of transferase activity** | 146 | 1.39 | 0.088 | 0.21 | 0.018 | 3'UTR |
| **immune response-activating cell surface receptor signaling pathway** | 140 | 1.43 | 0.11 | 0.20 | 0.014 | Intronic |
| **embryo development ending in birth or egg hatching** | 110 | 1.28 | 0.11 | 0.21 | 0.021 | 3'UTR |
| **antigen processing and presentation of peptide or polysaccharide antigen via MHC class II** | 2157 | 1.46 | 0.022 | 0.19 | 0.0037 | Intronic |
| **antigen processing and presentation of peptide or polysaccharide antigen via MHC class II** | 341 | 1.34 | 0.051 | 0.19 | 0.0094 | 3'UTR |
| **positive regulation of regulatory T cell differentiation** | 162 | 1.73 | 0.082 | 0.16 | 0.016 | Intronic |
| **detection of bacterium** | 178 | 1.62 | 0.076 | 0.17 | 0.012 | Downstream |
| **heme export** | 317 | 1.19 | 0.067 | 0.20 | 0.011 | Intronic |
| **regulation of microtubule polymerization** | 1198 | 1.33 | 0.031 | 0.19 | 0.0051 | Intronic |
| **negative regulation of smoothened signaling pathway involved in ventral spinal cord patterning** | 395 | 1.26 | 0.063 | 0.19 | 0.011 | Intronic |
| **bronchus morphogenesis** | 395 | 1.26 | 0.063 | 0.19 | 0.011 | Intronic |
| **defense response to tumor cell** | 121 | 1.60 | 0.10 | 0.16 | 0.017 | Intronic |
| **gamma-delta T cell activation** | 369 | 1.45 | 0.064 | 0.18 | 0.0087 | Intronic |

Note: nvar: number of SNPs in the domain; ave_k: average of GERP++ k per SNP; se_k: standard error of ave_k; ave_pi: average of nucleotide diversity per SNP; se_pi: standard error of ave_pi.

### Table D. Highly expressed miRNAs are generally conserved across species and have lower diversity in CHARGE WGS participants

| Expression Levels | miRNA conservation^a^ | | | π (x 1000) in miRNA precursors | | | π (x 1000) in mature miRNAs | | |
| --- | --- | --- | --- | --- | --- | --- | --- | --- | --- |
|  | # of conserved  miRNAs | # of non-conserved  miRNAs | Ratio of Conserved /  Non-conserved | Median | Mean | sd | Median | Mean | Sd |
| High | 188 | 65 | 2.89 | 0 | 0.379 | 1.39 | 0 | 0.311 | 2.45 |
| Median | 83 | 294 | 0.28 | 0 | 0.681 | 1.92 | 0 | 0.570 | 2.80 |
| Low | 48 | 801 | 0.06 | 0.0092 | 0.737 | 1.90 | 0 | 0.551 | 2.94 |
| Total miRNAs | 319 | 1160 | 0.28 | 0 | 0.662 | 1.83 | 0 | 0.493 | 2.75 |

^a^ Conserved miRNA means the first 20 nucleotide of a mature miRNA is conserved between human and at least one non-primate species.

### Table E. 42 mutations re-captured in this study are located in mature miRNAs and are segregating at intermediate to high frequencies (derived allele frequency >5% in the CHARGE WGS participants).

| **Chr.** | **Position**  **of SNP** | **Ancestral**  **allele** | **Derived**  **allele** | **Frequency of**  **derived allele** | **Mature**  **MiRNA** |
| --- | --- | --- | --- | --- | --- |
| 1 | 54519800 | A | G | 0.979 | hsa-miR-4781-3p |
| 1 | 67094171 | G | A | 0.161 | hsa-miR-3117-3p |
| 1 | 98510847 | T | C | 0.977 | hsa-miR-2682-3p |
| 1 | 2.28E+08 | C | T | 0.085 | hsa-miR-3620-5p |
| 2 | 64567916 | C | G | 0.281 | hsa-miR-4433-5p |
| 2 | 1.03E+08 | C | T | 0.084 | hsa-miR-4772-5p |
| 2 | 1.61E+08 | G | A | 0.082 | hsa-miR-4785 |
| 2 | 1.61E+08 | C | T | 0.124 | hsa-miR-4785 |
| 5 | 54466544 | A | G | 0.104 | hsa-miR-449b-5p |
| 5 | 54468124 | A | T | 0.323 | hsa-miR-449c-3p |
| 5 | 72174432 | G | C | 0.146 | hsa-miR-4804-5p |
| 5 | 1.51E+08 | C | T | 0.087 | hsa-miR-6499-5p |
| 5 | 1.6E+08 | G | C | 0.235 | hsa-miR-146a-3p |
| 5 | 1.69E+08 | C | T | 0.063 | hsa-miR-585 |
| 6 | 1.2E+08 | G | A | 0.227 | hsa-miR-3144-3p |
| 7 | 1.02E+08 | G | A | 0.133 | hsa-miR-5090 |
| 7 | 1.02E+08 | G | A | 0.457 | hsa-miR-4467 |
| 8 | 1765425 | T | C | 0.101 | hsa-miR-596 |
| 8 | 27559214 | G | A | 0.221 | hsa-miR-3622b-3p |
| 8 | 27559214 | G | A | 0.221 | hsa-miR-3622a-5p |
| 9 | 18573360 | G | A | 0.323 | hsa-miR-3152-3p |
| 10 | 29891260 | C | T | 0.272 | hsa-miR-938 |
| 10 | 1.03E+08 | G | C | 0.804 | hsa-miR-608 |
| 10 | 1.06E+08 | G | A | 0.302 | hsa-miR-4482-5p |
| 11 | 79133220 | T | C | 0.175 | hsa-miR-5579-3p |
| 12 | 26026988 | G | A | 0.13 | hsa-miR-4302 |
| 12 | 54385599 | C | T | 0.393 | hsa-miR-196a-3p |
| 12 | 94955585 | T | C | 0.083 | hsa-miR-5700 |
| 12 | 1.05E+08 | G | A | 0.27 | hsa-miR-3922-5p |
| 12 | 1.2E+08 | C | T | 0.966 | hsa-miR-1178-5p |
| 14 | 23426182 | C | A | 0.518 | hsa-miR-4707-3p |
| 14 | 1.02E+08 | A | G | 0.476 | hsa-miR-412 |
| 15 | 70371778 | A | G | 0.999 | hsa-miR-629-5p |
| 15 | 75081078 | G | A | 0.657 | hsa-miR-4513 |
| 16 | 81644970 | T | C | 0.121 | hsa-miR-6504-5p |
| 17 | 6558768 | C | T | 0.468 | hsa-miR-4520a-3p |
| 17 | 6558768 | C | T | 0.468 | hsa-miR-4520b-5p |
| 19 | 804959 | C | T | 0.098 | hsa-miR-4745-5p |
| 19 | 8454236 | A | G | 0.304 | hsa-miR-4999-5p |
| 20 | 33578251 | A | G | 0.206 | hsa-miR-499a-3p |
| 20 | 33578251 | A | G | 0.206 | hsa-miR-499b-5p |
| 20 | 58883605 | T | G | 0.078 | hsa-miR-646 |

# Supplementary Figures

### Figure A. Heterozygous concordance when comparing SNPs from WGS and WECS data.

Concordance rates of heterozygote genotypes of SNPs belong to different MAF bins. Each point is an average of 100 random subsampling from 886 individuals (total sample).


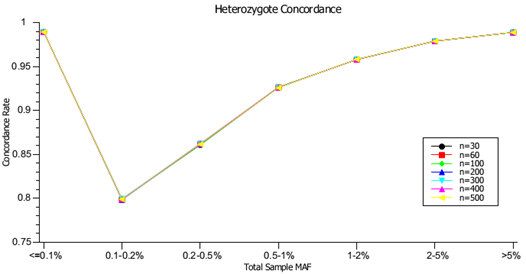


### Figure B. Site Frequency Spectrum (SFS) of the CHARGE WGS data compared to published demographic models.

a) comparison to Nelson *et al.* (with growth, dark blue) and Nelson *et al* without the last epoch of growth (no growth, brown). b) comparison with Tennessen *et al* (with growth, dark blue) and Tennessen *et al*  without the last epoch of growth (no growth, brown). In both panels, the proportions of all possible derived allele counts, ranging from 1 to 2n-1, sum up to 1, although only those for 1 through 15 are represented. Simulated SFS are based on 10,000 simulations. Sample size in all simulations matches the CHARGE WGS data sample size (923 individuals) used to derive the SFS. This sample size represents the full CHARGE WGS sample after excluding 39 individuals that showed a lack of homogeneity on the PCA.


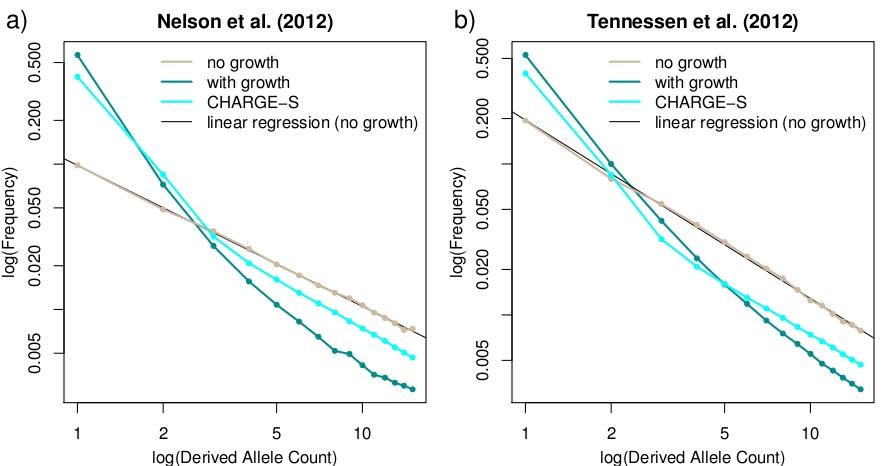


### Figure C. Distribution of the number of disease-causing alleles an individual carries in 962 CHARGE WGS participants.

Diseasing-causing alleles were defined as those recorded in HGMD with a “Variant_class” of “DM”. On average each individual carries 21.37 (*sd* =4.47) disease-causing alleles.

### Figure D. Principal components of genetic variation in CHARGE WGS participants estimated from (a) common variants (minor allele frequency > 5%) and (b) rare variants (minor allele frequency between 0.5-5%).

In (a) PC’s 1 and 2 account for 0.29% and 0.15% of the total variance, respectively. In (b) PC’s 1 and 2 account for 0.21% and 0.13% of the total variance, respectively. Coordinates on PC 1 have been inverted so as to be comparable to Figure S4a.

**(a)**


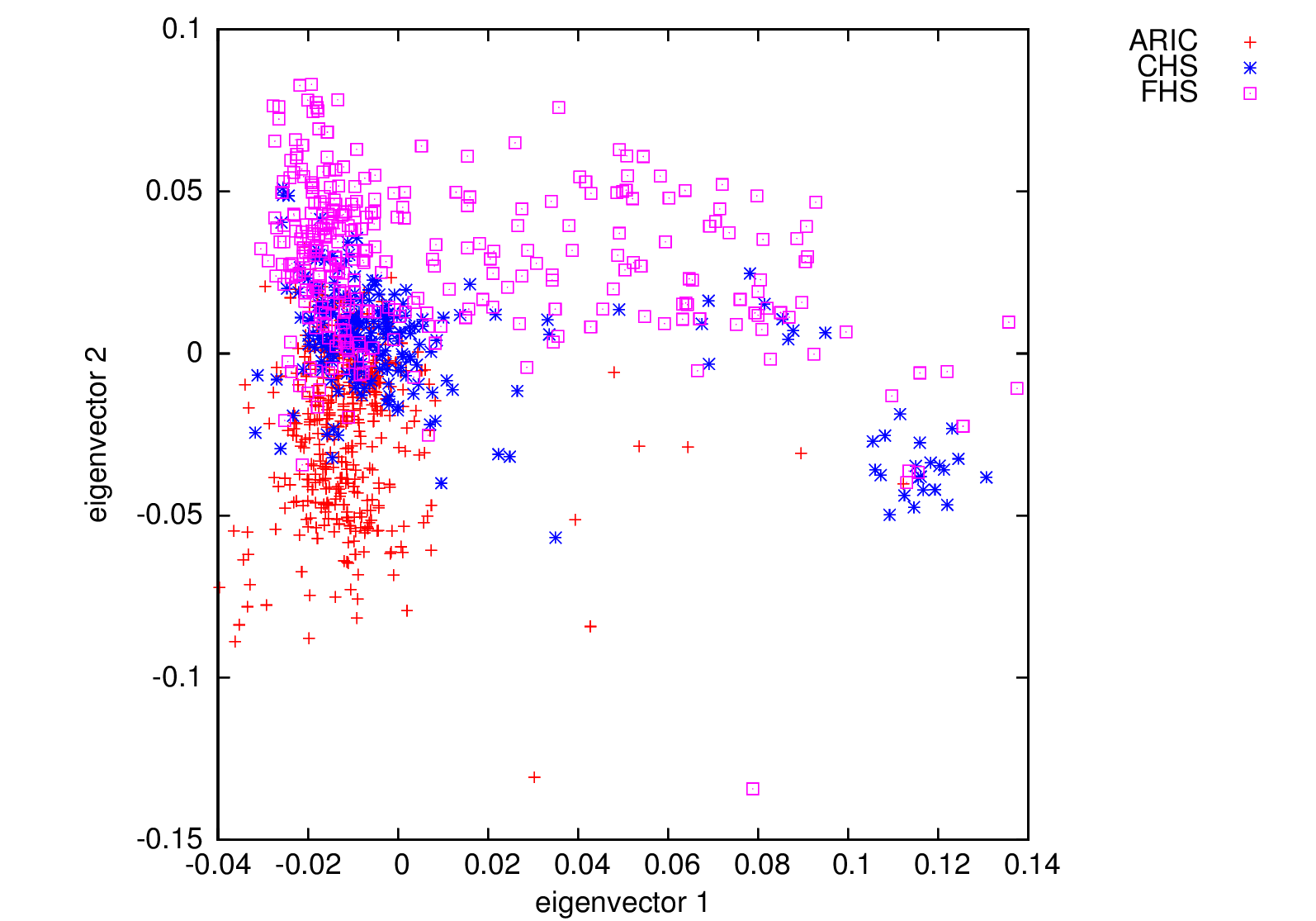


**(b)**


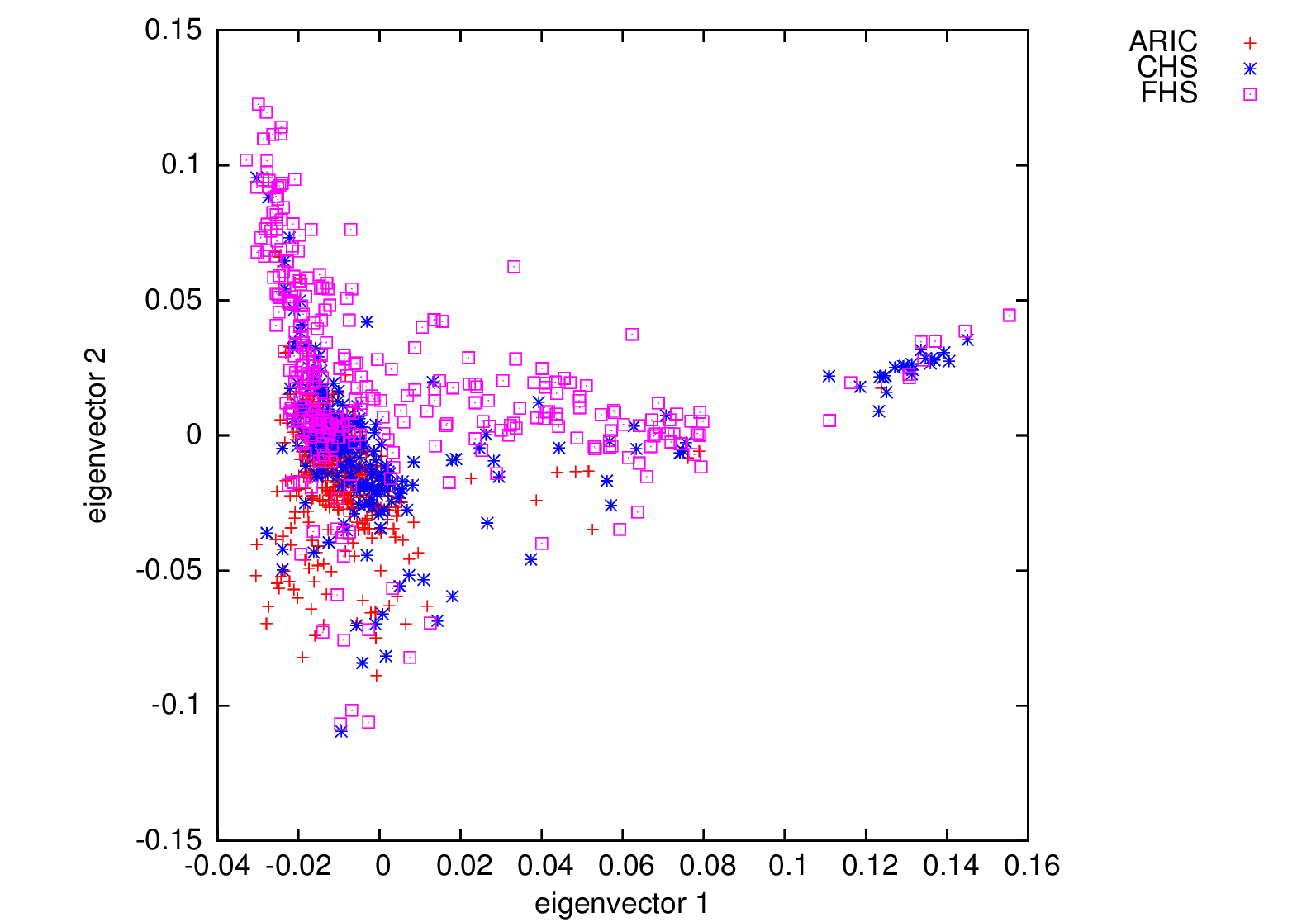


### Figure E. Principal components of genetic variation in HGDP participants with European or Middle Eastern ancestry with CHARGE WGS participants projected onto the PCs.

Thirty-one individuals labeled 'OutlierME' were identified as outliers in the whole genome rare-variant PCA analysis and appear to share recent common ancestry with Middle Eastern reference populations. PCs 1 and 2 account for 1.4 and 1.2% of the total variance, respectively.


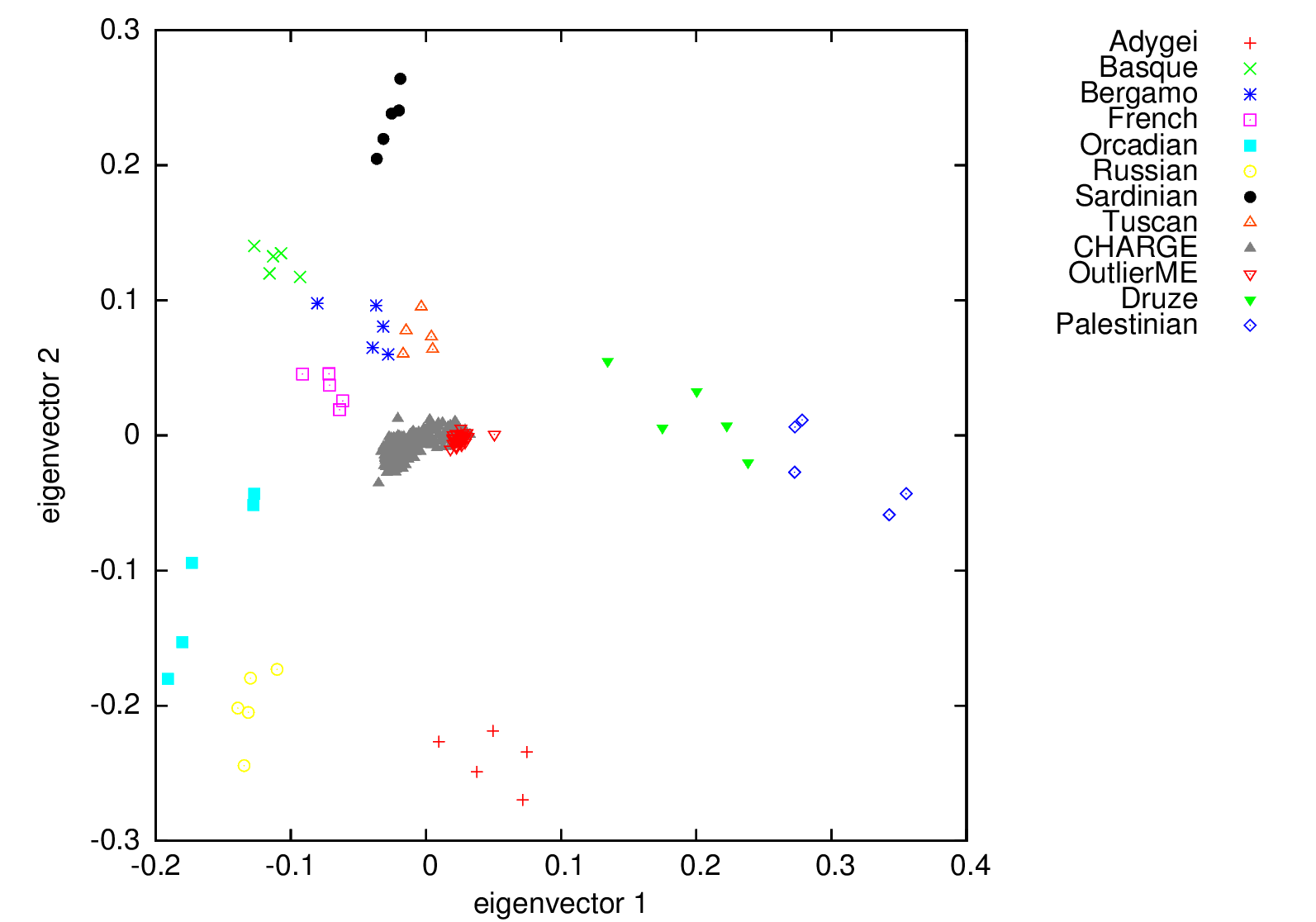


### Figure F. Principal components of genetic variation in HGDP participants with European or East Asian ancestry with CHARGE WGS participants projected onto the PCs.

Eight outlier individuals labeled 'OutlierEA' appear to share recent common ancestry with East Asian reference populations. PCs 1 and 2 account for 4.2 and 1.1% of the total variance, respectively.


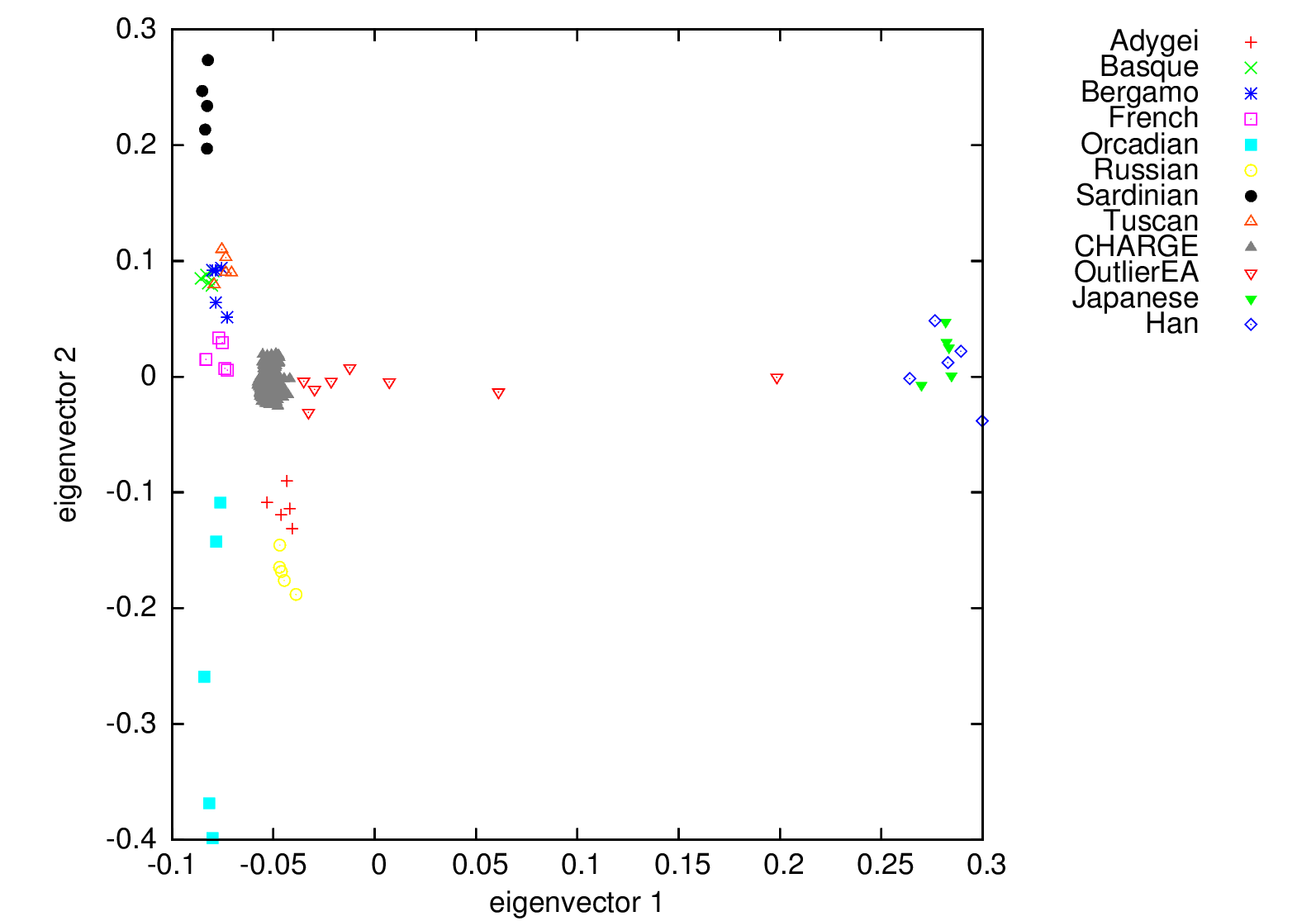


### Figure G. Four diversity measures of 500 bp sliding windows and iHS scores across 22 autosomes.

**Blue**: nucleotide diversity,(*π*); **Green**: Watterson’s *θ*; **Orange**: number of haplotypes; **Purple**: low bound of the number of recombination events; **Red**: iHS score.


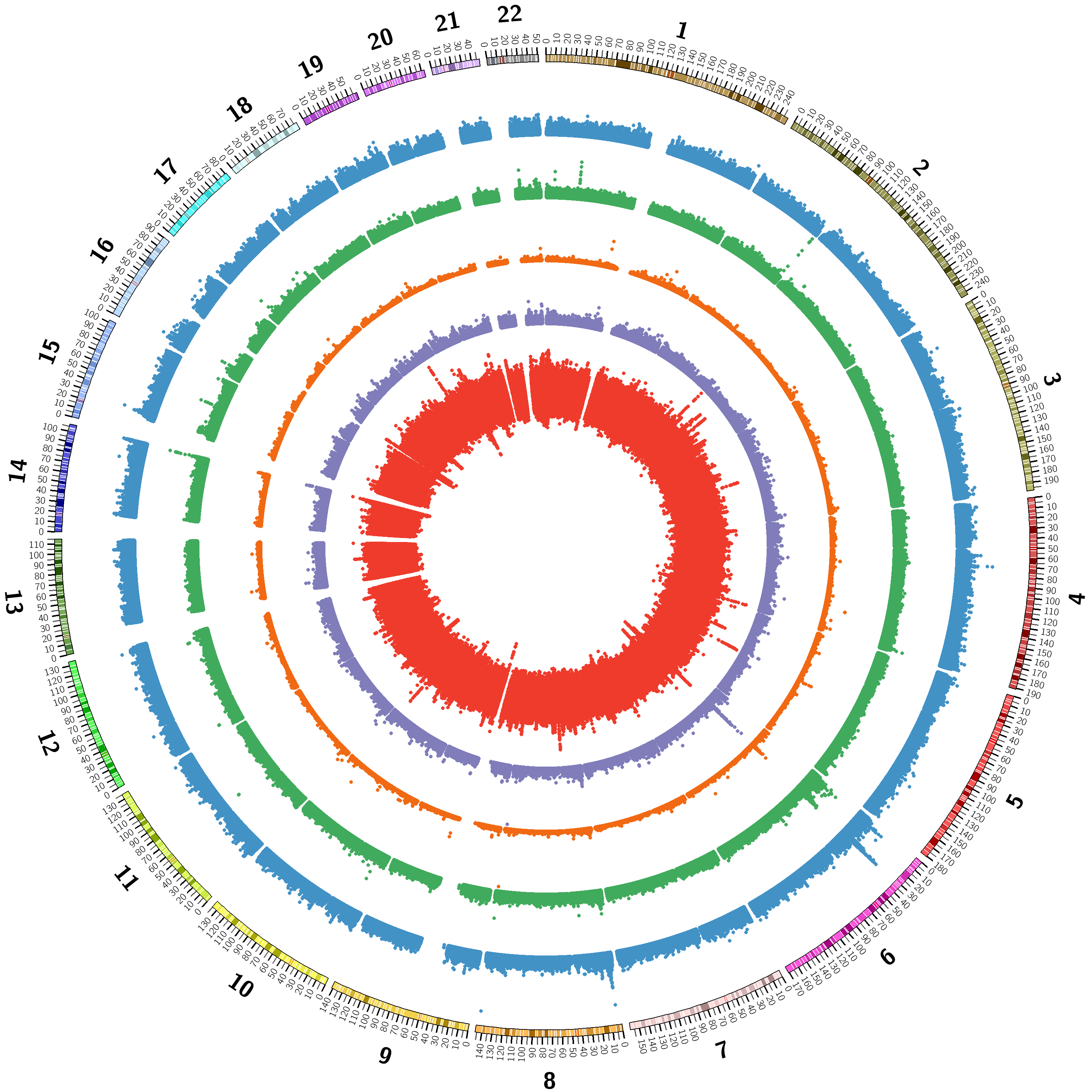


### Figure H. Enrichment of SNPs residing in regulatory regions in the group of SNPs showing deficiency of homozygote of minor allele.


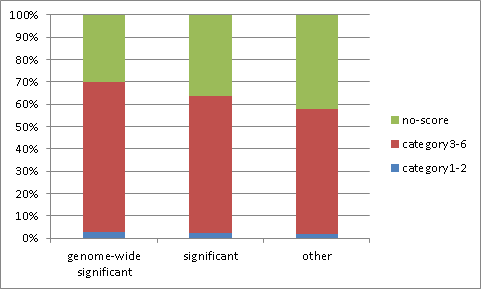


### Figure I. The distributions of derived allele frequencies (DAF) in miRNA precursor, mature miRNA and seed regions.

**A** is the distributions of derived allele frequencies (DAF) in all the miRNA loci. **B** is the distributions of derived allele frequencies (DAF) in the evolutionarily-conserved miRNA loci. For comparisons, the expected allele frequencies under neutral evolution of a Fisher-Wright population is plotted in both **A** and **B**. DAF of the introns of the protein-coding genes are also provided in both figures. The *x*-axis is the number of chromosomes carrying the derived mutations out of 1924 chromosomes (all the 962 participants were considered) and the *y*-axis is the percentage of the segregating sites for that mutation.


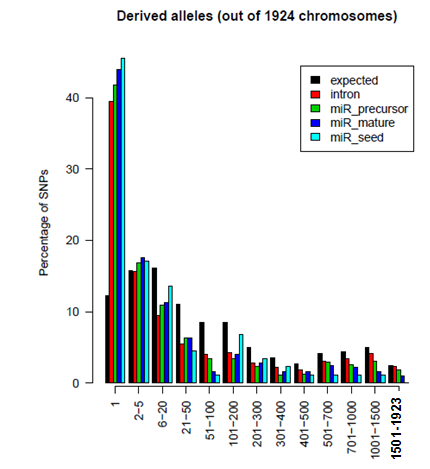


A


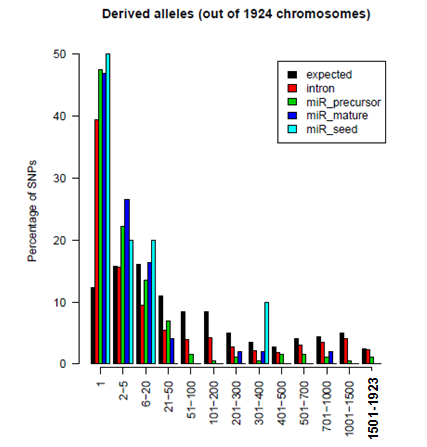


**B**

### Figure J. Distributions of derived allele frequencies (DAF) in 3’ UTRs and miRNA target sites.

For comparisons, the expected allele frequencies under neutral evolution of a Fisher-Wright population is plotted. DAF of the introns of the protein-coding genes are also provided. The *x*-axis is the number of chromosomes carrying the derived mutations out of 1924 chromosomes (all of the 962 participates are included) and the *y*-axis is the percentage of the segregating sites for that mutation. **(a)** the target sites of the conserved miRNAs that are identified with conservation criteria of miRNA:target pairing. Higher PCT score means higher stringency criteria. **(b)** the target sites of the conserved miRNAs that are identified with the context score (CS) of the miRNA pairing. Smaller context scores mean the target sites have high probability to be regulated by miRNAs.

a.


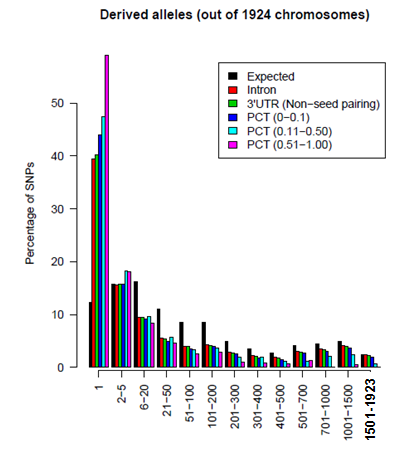


b.


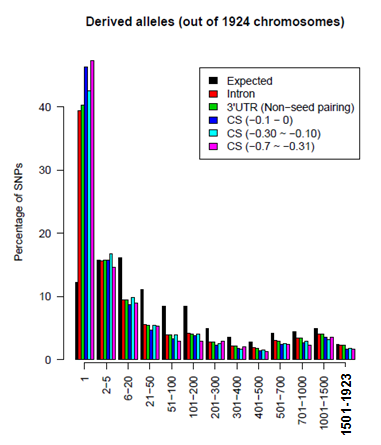


### Figure K. Distributions of derived allele frequencies (DAF) in introns and exons of lincRNAs, piRNAs and introns of coding regions.

The DAF analysis suggests piRNAs and lincRNAs evolving neutrally in the human populations.


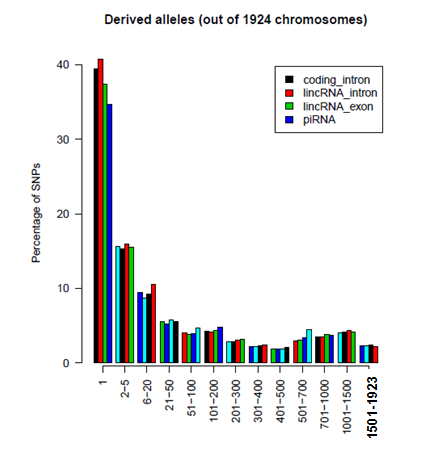

Supplement: S1 File — Fig. B, Site Frequency Spectrum (SFS) of the CHARGE WGS data compared to published demographic models. Fig. C, Distribution of the number of disease-causing alleles an individual carries in 962 CHARGE WGS participants. Fig. D, Principal components of genetic variation in CHARGE WGS participants estimated from (a) common variants (minor allele frequency > 5%) and (b) rare variants (minor allele frequency between 0.5–5%). Fig. E, Principal components of genetic variation in HGDP participants with European or Middle Eastern ancestry with CHARGE WGS participants projected onto the PCs. Fig. F, Principal components of genetic variation in HGDP participants with European or East Asian ancestry with CHARGE WGS participants projected onto the PCs. Fig. G, Four diversity measures of 500 bp sliding windows and iHS scores across 22 autosomes. Fig. H, Enrichment of SNPs residing in regulatory regions in the group of SNPs showing deficiency of homozygote of minor allele. Fig. I, The distributions of derived allele frequencies (DAF) in miRNA precursor, mature miRNA and seed regions. Fig. J, Distributions of derived allele frequencies (DAF) in 3’ UTRs and miRNA target sites. Fig. K, Distributions of derived allele frequencies (DAF) in introns and exons of lincRNAs, piRNAs and introns of coding regions. Table A, SNP calling quality summary. Table B, Top 20 domains with both low diversity and low divergence. Table C, Top 20 domains with both high diversity and high divergence. Table D, Highly expressed miRNAs are generally conserved across species and have lower diversity in CHARGE WGS participants. Table E, 42 mutations re-captured in this study are located in mature miRNAs and are segregating at intermediate to high frequencies (derived allele frequency >5% in the CHARGE WGS participants). (DOC) [file pone.0121644.s001.doc]
